# Evidence for Ubiquitin-Regulated Nuclear and Subnuclear Trafficking among *Paramyxovirinae* Matrix Proteins

**DOI:** 10.1371/journal.ppat.1004739

**Published:** 2015-03-17

**Authors:** Mickey Pentecost, Ajay A. Vashisht, Talia Lester, Tim Voros, Shannon M. Beaty, Arnold Park, Yao E. Wang, Tatyana E Yun, Alexander N. Freiberg, James A. Wohlschlegel, Benhur Lee

**Affiliations:** 1 Department of Microbiology, Immunology, and Molecular Genetics, David Geffen School of Medicine, University of California Los Angeles, Los Angeles, California, United States of America; 2 Department of Biological Chemistry, David Geffen School of Medicine, University of California Los Angeles, Los Angeles, California, United States of America; 3 Department of Microbiology, Icahn School of Medicine at Mount Sinai, New York, New York, United States of America; 4 Department of Pathology, University of Texas Medical Branch, Galveston, Texas, United States of America; University of Pittsburgh, UNITED STATES

## Abstract

The paramyxovirus matrix (M) protein is a molecular scaffold required for viral morphogenesis and budding at the plasma membrane. Transient nuclear residence of some M proteins hints at non-structural roles. However, little is known regarding the mechanisms that regulate the nuclear sojourn. Previously, we found that the nuclear-cytoplasmic trafficking of Nipah virus M (NiV-M) is a prerequisite for budding, and is regulated by a bipartite nuclear localization signal (NLS_bp_), a leucine-rich nuclear export signal (NES), and monoubiquitination of the K258 residue within the NLSbp itself (NLS_bp_-lysine). To define whether the sequence determinants of nuclear trafficking identified in NiV-M are common among other *Paramyxovirinae* M proteins, we generated the homologous NES and NLS_bp_-lysine mutations in M proteins from the five major *Paramyxovirinae* genera. Using quantitative 3D confocal microscopy, we determined that the NES and NLS_bp_-lysine are required for the efficient nuclear export of the M proteins of Nipah virus, Hendra virus, Sendai virus, and Mumps virus. Pharmacological depletion of free ubiquitin or mutation of the conserved NLS_bp_-lysine to an arginine, which inhibits M ubiquitination, also results in nuclear and nucleolar retention of these M proteins. Recombinant Sendai virus (rSeV-eGFP) bearing the NES or NLS_bp_-lysine M mutants rescued at similar efficiencies to wild type. However, foci of cells expressing the M mutants displayed marked fusogenicity in contrast to wild type, and infection did not spread. Recombinant Mumps virus (rMuV-eGFP) bearing the homologous mutations showed similar defects in viral morphogenesis. Finally, shotgun proteomics experiments indicated that the interactomes of *Paramyxovirinae* M proteins are significantly enriched for components of the nuclear pore complex, nuclear transport receptors, and nucleolar proteins. We then synthesize our functional and proteomics data to propose a working model for the ubiquitin-regulated nuclear-cytoplasmic trafficking of cognate paramyxovirus M proteins that show a consistent nuclear trafficking phenotype.

## Author Summary

Elucidating virus-cell interactions is fundamental to understanding viral replication and identifying targets for therapeutic control of viral infection. Paramyxoviruses include human and animal pathogens of medical and agricultural significance. Their matrix (M) structural protein organizes virion assembly at the plasma membrane and mediates viral budding. While nuclear localization of M proteins has been described for some paramyxoviruses, the underlying mechanisms of nuclear trafficking and the biological relevance of this observation have remained largely unexamined. Through comparative analyses of M proteins across five *Paramyxovirinae* genera, we identify M proteins from at least three genera that exhibit similar nuclear trafficking phenotypes regulated by an NLS_bp_ as well as an NES sequence within M that may mediate the interaction of M with host nuclear transport receptors. Additionally, a conserved lysine within the NLS_bp_ of some M proteins is required for nuclear export by regulating M ubiquitination. Sendai virus engineered to express a ubiquitination-defective M does not produce infectious virus but instead displays extensive cell-cell fusion while M is retained in the nucleolus. Thus, some *Paramyxovirinae* M proteins undergo regulated and active nuclear and subnuclear transport, a prerequisite for viral morphogenesis, which also suggests yet to be discovered roles for M in the nucleus.

## Introduction

Paramyxoviruses include pathogens of global medical and agricultural concern. These viruses occupy broad ecological niches infecting a wide range of hosts including mammals, reptiles, birds and fish, and they cause diverse outcomes ranging from asymptomatic infection to lethal disease. Measles virus (MeV), mumps virus (MuV), the human parainfluenza viruses (hPIVs), respiratory syncytial virus (RSV), and human metapneumoviruses remain significant causes of human morbidity and mortality [1]. Animal pathogens, such as Newcastle disease virus (NDV) and the recently eradicated Rinderpest virus [2], have caused significant rates of lethal disease in birds and cattle, respectively. The newly emergent zoonotic paramyxoviruses Nipah virus (NiV) and Hendra virus (HeV) are among the most deadly known pathogens, showing case-fatality rates in excess of 70% in humans, and are classified as biosafety level 4 pathogens due to the absence of vaccines or therapeutics approved for human use [3–6].

Paramyxoviruses are released as enveloped virions from the host cell plasma membrane. Virions are ~150–300 nm in diameter and are spherical, pleomorphic or filamentous in shape, depending on the virus and the producer cell-type. The non-segmented, single-strand, negative-sense RNA genomes of paramyxoviruses consist of six principal genes: nucleocapsid (N), phosphoprotein (P), matrix (M), fusion (F) and attachment (HN, H or G) glycoproteins, and polymerase (L) [1,5,7]. The attachment and fusion glycoproteins mediate binding to sialic acid moieties or to specific protein receptors on the cell surface and the fusion of the viral envelope with the host cell plasma membrane [8–10]. Within the virion, the ribonucleoprotein (RNP) consists of the RNA-dependent RNA polymerase complex formed by P and L associated with the N-encapsidated RNA genome. L is required for viral RNA synthesis during viral replication [1,5].

M is the primary viral structural protein [1,5,7]. A number of studies have found that M proteins oligomerize, bind lipids, and form a grid-like array on the inner surface of the viral membrane (_((((xxx))))_)[7,11–25]. M proteins can serve as a molecular scaffold by interacting with the cytoplasmic tails of the transmembrane glycoproteins and the RNP via N [7,17,25–35]. Many paramyxoviral M proteins (NiV-M, MeV-M, NDV-M, SeV-M, and hPIV1-M) can drive viral budding and form virus-like particles (VLPs) in the absence of other viral components [13,31,36–42], albeit with varying efficiencies. However, the budding of some others (PIV5-M and MuV-M) requires coexpression of N and/or the envelope glycoproteins [43,44]. MeV and SeV engineered with budding-defective or deleted M proteins have been found to have severe defects in viral replication [45–47].

Although paramyxoviruses are classic cytoplasmic replicating viruses, some paramyxoviral M proteins have been observed to traffic through the nucleus. For example, SeV-M, NDV-M and RSV-M can be detected in the nucleus at early stages of infection [48–53]. These findings suggest that paramyxoviral M proteins may perform roles beyond viral assembly at the plasma membrane. However, with the exception of RSV, which belongs to the *Pneumovirinae* subfamily, the cell biology of M protein nuclear trafficking has not been examined in a systematic fashion for most *Paramyxovirinae* subfamily members. We previously found that NiV-M translocates to the nucleus at early stages of infection. The high homology between NiV-M and HeV-M (~90% amino acid identity) suggests that HeV-M also localizes to the nucleus, and it was recently found that overexpression of ANP32B, a nuclear protein, results in nuclear accumulation of HeV-M and NiV-M [54]. We have shown that nuclear-cytoplasmic trafficking of NiV-M is mediated by a classical bipartite nuclear localization signal (NLS_bp_), homologous to NDV-M’s NLS_bp_, and a leucine-rich nuclear export signal (NES) [39,48]. We further demonstrated that nuclear trafficking is regulated by ubiquitination, presumably on a conserved lysine residue (K258) located within the NLS_bp_ of NiV-M (^244^RR-X10-RR**K** ^258^). The K258A mutant is defective in nuclear import, while the K258R mutant retains a functional NLS but is defective in nuclear export; both mutants have decreased levels of ubiquitination and have budding defects [39].

The canonical NES and NLS_bp_ that we functionally characterized in NiV-M are highly conserved across most, if not all members of the *Paramyxovirinae*. Therefore, it is important to resolve whether ubiquitin-dependent nuclear-cytoplasmic trafficking of M is unique to NiV, or to what extent other members of the subfamily also exhibit a nuclear-trafficking phenotype. Uncovering the mechanisms that govern paramyxovirus M protein trafficking has direct bearing on the fundamental biology of paramyxoviral replication, and may reveal host-dependent pathways and factors that can be exploited for antiviral strategies. Here, we specifically analyze ubiquitin-dependent nuclear-cytoplasmic trafficking of M proteins across representative viruses from all five major genera of *Paramyxovirinae* (*Respirovirus*, *Rubulavirus*, *Morbillivirus*, *Henipavirus*, and *Avulavirus*). We use a panoply of methods including quantitative 3D confocal microscopy analysis of M nuclear localization, bimolecular fluorescence complementation (BiFC) assays of M ubiquitination, and introduction of M mutations into live recombinant viruses with the use of reverse genetics. Our findings demonstrate that ubiquitination of M, regulated by a lysine within the second basic patch of the NLS_bp_, critically modulates the subnuclear and nuclear-cytoplasmic trafficking of M proteins from prototypic viruses of the *Henipavirus*, *Rubulavirus* and *Respirovirus* genera. Proteomic identification of nuclear transport receptors and nuclear pore complex components that copurify with paramyxoviral M proteins further supports a model for active transport of M in and out of the nucleus, and also hints at possible non-structural functions of M proteins.

## Results

### Nuclear export of some *Paramyxovirinae* matrix proteins is regulated by the ubiquitin-proteasome system

**Fig 1:**
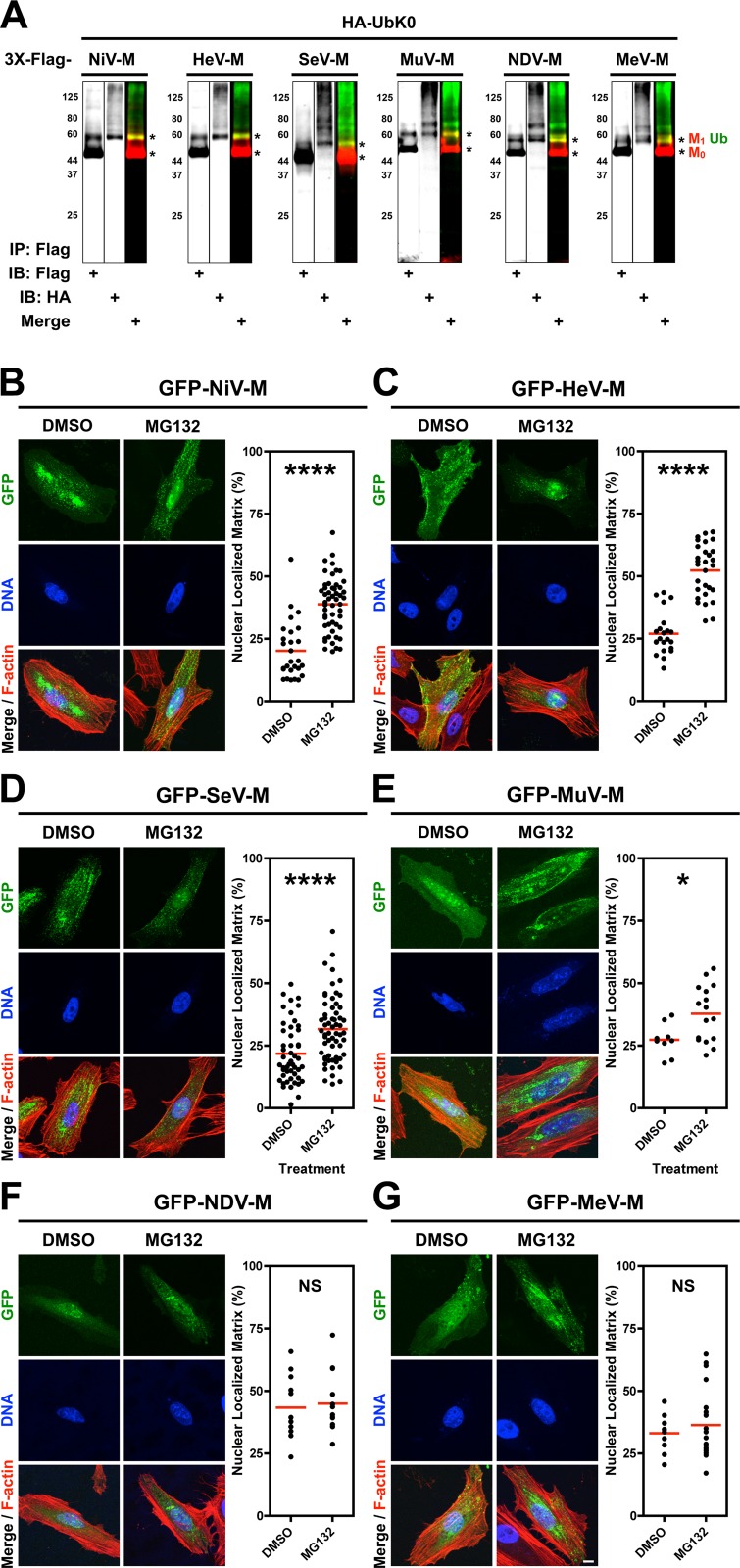
Analysis of the ubiquitin-regulated nuclear export of matrix proteins from five *Paramyxovirinae* genera. **(A)** Ubiquitination of M proteins. HEK 293T cells were cotransfected with HA-UbK0 and 3X-Flag tagged NiV-M, HeV-M, SeV-M, MuV-M, NDV-M, or MeV-M. After 24h, 3X-Flag-tagged-M was immunoprecipitated, and M and its ubiquitinated species were detected by immunoblotting against Flag and HA, respectively. **(B-G)** 3D confocal analysis of M nuclear localization in cells depleted of free ubiquitin. Extended Focus (maximum intensity projection) view of 3D confocal micrographs of HeLa cells transfected with GFP-tagged **(B)** NiV-M, **(C)** HeV-M, **(D)** SeV-M, **(E)** MuV-M, **(F)** NDV-M, or **(G)** MeV-M. At 16 h post-transfection, cells were treated with 50 μM MG132/0.5% DMSO or 0.5% DMSO for 8h. Cells were counterstained with DAPI to visualize nuclear DNA, blue, and fluorescent phalloidin to visualize the F-actin cytoskeleton, red. Scale bar 10 μm. In the corresponding graphs, the nuclear M fluorescence per cell was quantified from 3D-reconstructed confocal micrographs. *p<0.05; ****p<0.0001; NS, not significant by Student's t-test.

Since the nuclear-cytoplasmic trafficking of the Nipah virus matrix protein (NiV-M) is regulated by its monoubiquitination [39], we wondered whether the ubiquitin-proteasome system similarly regulates the nuclear sojourn of other *Paramyxovirinae* M proteins. We cloned 3X-Flag- and GFP-tagged-M from prototypical members of the five *Paramyxovirinae* genera: NiV-M (genus *Henipavirus*), Hendra virus M (HeV-M, genus *Henipavirus*), Sendai virus M (SeV-M, genus *Respirovirus*), Mumps virus M (MuV-M, genus *Rubulavirus*), Newcastle disease virus M (NDV-M, genus *Avulavirus*), and Measles virus M (MeV-M, genus *Morbillivirus*). To biochemically detect ubiquitination of M proteins, we cotransfected HEK 293T cells with HA-UbK0 and each of 3X-Flag-tagged NiV-M, HeV-M, SeV-M, MuV-M, NDV-M or MeV-M [55]. HA-UbK0 functions as a ubiquitin (Ub) chain terminator or as monoubiquitin because all lysines have been mutated to arginines. We used this construct to visualize discrete ubiquitin bands and to determine if matrix proteins can be monoubiquitinated since this posttranslational modification can regulate the function of proteins without promoting proteasome-dependent protein degradation [56]. Cell lysates were subjected to anti-Flag immunoprecipitation (IP) and immunoblots were simultaneously probed with anti-HA and anti-Flag antibodies. As shown in Fig. 1A, for all the M proteins the majority of M is unmodified (M_0_) at steady state. However, a detectable minority of M (M_1_) is size-shifted by the molecular weight of at least one ubiquitin monomer (Ub, ~8.5 kDa) (Fig. 1A, merge). These results indicate that all 3X-Flag-tagged M proteins investigated are ubiquitin substrates.

We have found that proteasome inhibition results in nuclear retention of NiV-M in transfected and in NiV-infected cells (S1 Fig.) [39]. Proteasome inhibition stabilizes polyubiquitinated proteins and depletes the cellular levels of free ubiquitin available for conjugation [57–62]. To determine whether ubiquitination is involved in the nuclear export of the other *Paramyxovirinae* M proteins, we treated GFP-M-expressing HeLa cells with the proteasome inhibitor MG132 (Fig. 1B-G). We used quantitative 3D confocal microscopy to characterize the subcellular localization of M. The cells were counterstained with DAPI to visualize nuclei, and with fluorescent phalloidin to visualize the entire cell, and the proportion of nuclear M was determined computationally as described in Materials and Methods. As with GFP-NiV-M, ubiquitin depletion via proteasome inhibition resulted in significant nuclear retention of GFP-tagged HeV-M, SeV-M and MuV-M (Fig. 1B-E) [39]. We further confirmed biochemically that MG132 reduces the direct conjugation of ubiquitin to 3X-Flag-tagged NiV-M, HeV-M, SeV-M, MuV-M, MeV-M and NDV-M by co-IP of each and HA-UbK0, as described above, with quantification of immunoblot band integrated intensities as described in Materials and Methods (S2 Fig.). The ubiquitination of the various M proteins were differentially sensitive to proteasome inhibition with NDV-M and MeV-M being the least and most sensitive to proteasome inhibition, respectively (S2E–F Fig.). For NDV-M (*Avulavirus*) and MeV-M (*Morbillivirus*), reduction of ubiquitin conjugation did not result in a nuclear retention phenotype under the conditions and cell type examined (Fig. 1F-G), suggesting there is no strict correlation between the degree of matrix ubiquitination *per se* and M nuclear localization. In contrast, the ubiquitin-proteasome system appears to regulate the nuclear-cytoplasmic trafficking of the *Henipavirus* (NiV-M, HeV-M), *Respirovirus* (SeV-M), and *Rubulavirus* (MuV-M) matrix proteins.

### Nuclear export of some *Paramyxovirinae* matrix proteins is regulated by a putative NES and a lysine within the NLS_bp_

**Fig 2:**
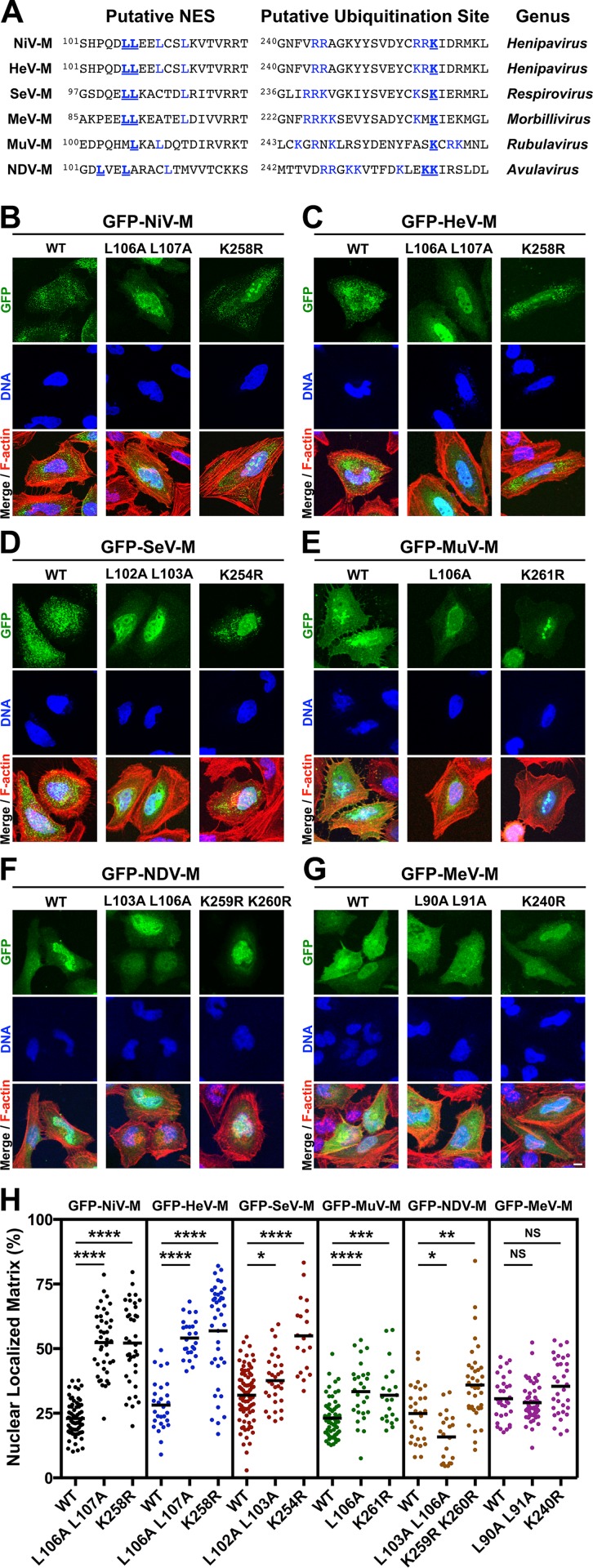
Mutational analysis of the role of a putative NES and a lysine within the NLS_bp_ in nuclear export of *Paramyxovirinae* matrix proteins. **(A)** Alignment of *Paramyxovirinae* M sequence motifs that correspond to NiV-M's leucine-rich NES and NLS_bp_, which contains a putative ubiquitinated lysine. Predicted critical residues are colored blue. Residues mutated in this study are also underlined in bold font. **(B-G)** Extended Focus (maximum intensity projection) views of 3D confocal micrographs of HeLa cells transfected with WT, or the indicated mutant GFP-tagged **(B)** NiV-M, **(C)** HeV-M, **(D)** SeV-M, **(E)** MuV-M, **(F)** NDV-M or **(G)** MeV-M. Cells were counterstained with DAPI to visualize nuclear DNA, blue, and fluorescent phalloidin to visualize the F-actin cytoskeleton, red. Scale bar 10 μm. **(H)** The amount of nuclear M fluorescence per cell was quantified from 3D reconstructed confocal micrographs. *p<0.05; **p<0.01; ***p<0.001; ****p<0.0001; NS, not significant by one-way ANOVA with Bonferroni adjustment for multiple comparisons.

We have shown that the nuclear-export of NiV-M is regulated by a leucine-rich nuclear export signal (NES) as well as by the K258 lysine residue located within the second basic patch of the bipartite nuclear localization signal (NLS_bp_; Fig. 2A, blue residues) [39]. A K258A mutation partially disrupts the NLS_bp_ and decreases nuclear localization of NiV-M, while a K258R mutation is unexpectedly retained in the nucleus despite having intact NES sequences and preservation of the positive charge necessary for NLSbp function. However, both mutants are impaired for ubiquitination [39]. Sequence alignment of M proteins indicates that a lysine is present in the homologously aligned position across the *Paramyxovirinae* genera. Thus, we hypothesized that this residue might be conserved for regulation of M ubiquitin-dependent nuclear export (Fig. 2A, bold and underlined blue residues). To interrogate our hypothesis, we mutated the NLS_bp_-lysine to an arginine in all M proteins studied and analyzed their subcellular localization by quantitative 3D confocal microcopy as described above (Fig. 2B-G, quantified in Fig. 2H). Since NDV-M contains another lysine adjacent to this position we mutated both (Fig. 2A, bold and underlined blue residues). A lysine to arginine mutation is expected to preserve the nuclear import function of the putative NLS_bp_, but prevents posttranslational modification at that position. As a comparison for nuclear retention, we also mutated the leucines that correspond to the NES of NiV-M within all M proteins (Fig. 2A, bold and underlined blue residues) [39].

Mutation of the NES of GFP-NiV-M (M_L106A L107A_) resulted in a significant increase in nuclear localization of the protein, which confirms our previous findings (Fig. 2B, 2H) [39]. Similarly, but to varying degrees, GFP-tagged HeV-M_L106A 107A_, SeV-M_L102A L103A_, and MuV-M_L106A_ also exhibited significantly increased nuclear retention compared to their respective wild type (WT) proteins (Fig. 2C-2E, 2H). In contrast, GFP-NDV-M_L103A L106A_ had an apparent nuclear exclusion phenotype (Fig. 2F, 2H) contrary to expectations, while the nuclear localization of GFP-MeV-M_L90A 191A_ was not significantly different than WT (Fig. 2G, 2H), indicating that these motifs are either redundant or non-functional in NDV-M and MeV-M.

Mutation of the NLS_bp_-lysine resulted in significantly enhanced nuclear localization of GFP-tagged NiV-M_K258R_, HeV-M_K258R_, SeV-M_K254R_, MuV-M_K261R_, and NDV-M_K259R K260R_, but not MeV-M_K240R_ (Fig. 2B-2G). These phenotypic differences are quantified in Fig. 2H. Note that the spread in the degree of nuclear localization for any given M mutant also emphasizes the need to score a sufficient number of cells by computationally defined volumetric criteria (see Materials and Methods) in order to obtain robust statistics from inherently variable cell biological data. Thus, each data point in Fig. 2H (as in Fig. 1B-G) represents reconstructed volumetric data from a single cell, acquired from ~20–30 confocal optical Z-stacks (at 0.3–0.5 μm/step) per cell. In contrast to the NLS_bp_-lysine-to-arginine mutations, our attempt to disrupt the NLS_bp_ consensus sequence through alanine substitutions in the second patch of basic residues (bp2) resulted in diffuse cytoplasmic localization of GFP-tagged NiV-M, HeV-M, SeV-M, and MuV-M (S3 Fig.). However, similar mutations did not appear to disrupt the localization of MeV-M or NDV-M (S3 Fig.) indicating that this motif does not function as the NLS_bp_ in MeV-M or that additional mutations are necessary to fully disrupt the function of the NLS_bp_ as has been previously shown for NDV-M [48].

### Ubiquitination of Nipah, Hendra, Sendai and Mumps matrix proteins is dynamically regulated by an NLS_bp_ lysine

**Fig 3:**
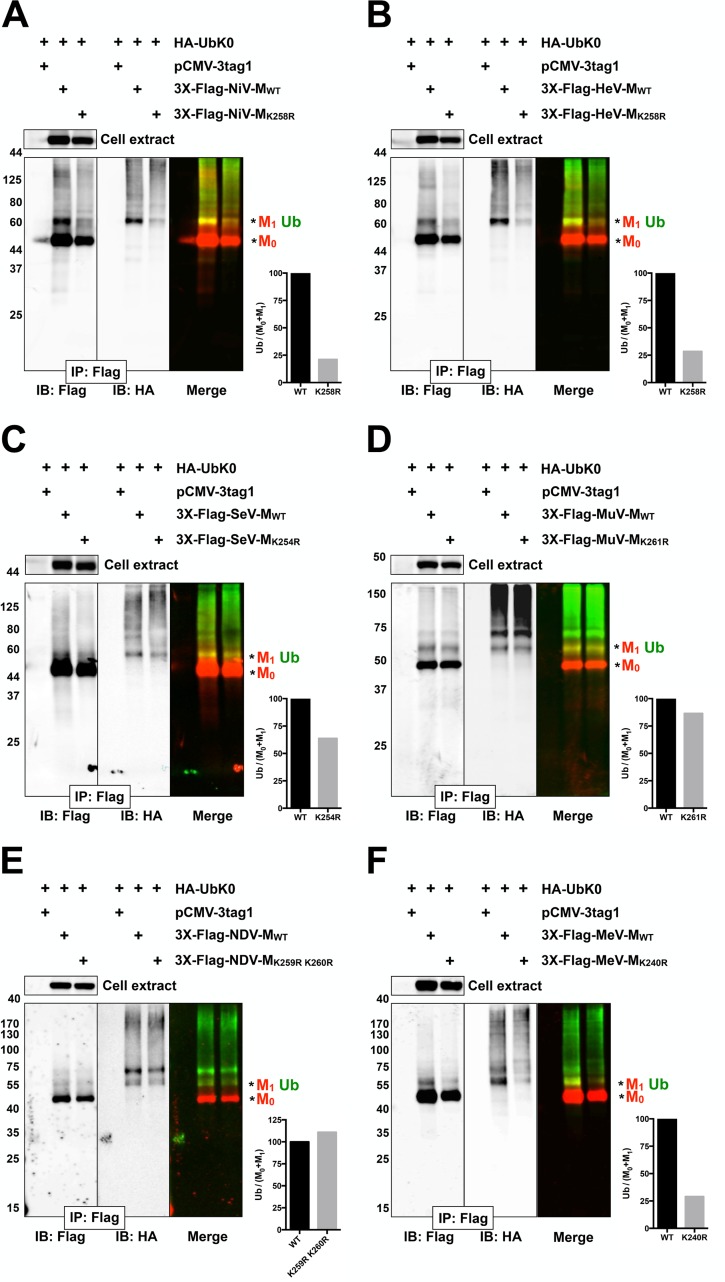
Biochemical analysis of *Paramyxovirinae* matrix ubiquitination regulated by a lysine within the NLS_bp_. **(A-F)** Immunoblot analysis of ubiquitination of the NLS_bp_-lysine mutants of NiV-M, HeV-M, SeV-M, MuV-M, NDV-M and MeV-M. HEK 293T cells were cotransfected with HA-UbK0 and the WT or the indicated NLS_bp_-lysine mutant 3X-Flag tagged **(A)** NiV-M, **(B)** HeV-M, **(C)** SeV-M, **(D)** MuV-M, **(E) NDV-M**, and **(F) MeV-M**. After 24h, 3X-Flag-tagged-M was immunoprecipitated, and M and its ubiquitinated species were detected by immunoblotting against Flag and HA, respectively. The background subtracted integrated fluorescence intensities of the monoubiquitin bands (Ub) normalized to total M (M_0_+M_1_) was determined using LI-COR Odyssey software.

Since the proteasome inhibitor MG132 inhibits the nuclear export of GFP-tagged NiV-M, HeV-M, SeV-M, and MuV-M (Fig. 1), we wanted to test whether the NLS_bp_-lysine that regulates their nuclear export (Fig. 2B-E) also regulates their ubiquitination. In Fig. 3, we first assessed the ability of 3X-Flag-tagged NiV-M_K258R_, HeV-M_K258R_, SeV-M_K254R,_ MuV-M_K261R_, MeV-M_K240R_ and NDV-M_K259R K260R_ to be ubiquitinated biochemically, via co-IP of 3X-Flag-tagged-M with HA-UbK0, as described above (Fig. 3A-D). Although NDV-M and MeV-M did not exhibit a ubiquitin-dependent nuclear trafficking phenotype (Fig. 1F-G), we included NDV-M_K259R K260R_ and MeV-M_K240R_ in this experiment since the ubiquitin conjugation of WT NDV-M and MeV-M was sensitive to MG132 inhibition, albeit to varying degrees (S2E–F Fig.). We controlled for protein abundance by normalizing the integrated intensity of the Ub band by the integrated intensity of the total M (Ub/M_0_+M_1_). Using this measure, 3X-Flag-tagged NiV-M_K258R_ (Fig. 3A), HeV-M_K258R_ (Fig. 3B) and MeV-M_K240R_ (Fig. 3E) exhibited the greatest reduction in relative monoubiquitination compared to the WT proteins (>70%), while 3X-Flag-tagged SeV-M_K254R_ (Fig. 3C) and MuV-M_K261R_ (Fig. 3D) showed only a modest to mild impairment in monoubiquitination (36% and ~15% reduction, respectively). 3X-Flag-tagged NDV-M_K259R K260R_ did not display reduced ubiquitination (Fig. 3F). Residual ubiquitination of the matrix mutants indicates that other lysines within the 3X-Flag-tagged M proteins are also targets of ubiquitin conjugation. Table 1 summarizes the results obtained thus far: although ubiquitinated species can be detected for all six matrix proteins examined (Fig. 1A, S2 Fig.), only NiV-M, HeV-M, SeV-M, and, MuV-M displayed a ubiquitin-dependent nuclear-cytoplasmic trafficking phenotype (Fig. 1B-1E, S2B–S2E Fig.) that was also dependent on a lysine in the NLS_bp_ (Fig. 2B-2E).

**Table 1:** Summary of Paramyxovirinae matrix protein nuclear trafficking phenotypes.

	Presence of ubiquitin conjugates (basal levels)	Ubiquitin conjugates decreased by MG132		Nuclear localization increased by MG132		Nuclear localization increased with NES mutation		Nuclear localization increased with NLS_bp2_ [K→R] mutation		NES and NLS_bp_ aligned at homologous positions	Nuclear exclusion increased with NLSbp2 [R/K,R/K,K→AAA] mutation (qualitative)	Ubiquitin conjugates decreased with NLS_bp2_ [K→R] mutation		Proteasome inhibition phenocopies nuclear and nucleolar phenotype of NLSbp2 [K→R] mutation	
	Fig. 1A (IP-IB)	S2 Fig. (IP-IB)		Fig. 1 (MIP)		Fig. 2 (MIP)		Fig. 2 (MIP)		Fig. 2A	S3 Fig. (MIP)	Fig. 3 (IP-IB)	Fig. 4 (BiFC)	Matrix only (XYZ)	Live virus (XYZ)
**NiV-M**	Yes	^++^	(S2B)	^+++^	(1B)	^++++^	(2B)	^++++^	(2B)	Yes	Yes	^+++^	^+++^	Yes (Fig. 5A)	Yes (S1 Fig.)
**HeV-M**	Yes	^++^	(S2C)	^+++^	(1C)	^+++^	(2C)	^++++^	(2C)	Yes	Yes	^++^	^+++^	Yes (Fig. 5B)	N.D.
**SeV-M**	Yes	^+++^	(S2D)	^++^	(1D)	^++^	(2D)	^+++^	(2D)	Yes	Yes	^+^	^++^	Yes (Fig. 5C)	Yes (Fig. 6 and 7)
**MuV-M**	Yes	^+++^	(S2E)	^+^	(1E)	^+^	(2E)	^+^	(2E)	Yes	Yes	^+/-^	^++^	Yes (Fig. 5D)	(S5 Fig.)***
**NDV-M**	Yes	^+^	(S2F)	-	(1F)	^(-)^ *	(2F)	^+^	(2F)	Yes	No**	^(-)^	N.D.	N.D.	N.D.
**MeV-M**	Yes	^+++^	(S2G)	-	(1G)	-	(2G)	-	(2G)	Yes	No**	^++^	N.D.	N.D.	N.D.

^(-)^,<0%;-, 0–10%;

^+/-^, 10–25%;

^+^, 25–50%;

^++^, 50–75%;

^+++^, 75–100%;

^++++^,>100%;

IP-IB, immunoprecipitation followed by immunoblot;

MIP, maximum intensity projection of 3D confocal micrographs;

BiFC, bimolecular fluorescence complementation; XYZ, XYZ planes view of 3D confocal micrographs;

N.D., not determined;

*, NES mutation gave unexpected increased nuclear exclusion rather than the expected increased nuclear localization (see Fig. 2H for quantification);

**, Subcellular localization phenotypes of the NES and the various NLSbp2 mutants for NDV-M and MeV-M are not congruent (compare S3 Fig. and Fig. 2F and 2G);

***, Recombinant MuV rescued with NES or NLSbp2 mutations. Defects in viral morphogenesis were observed but subcellular localization of MuV-M could not be determined due to a lack of specific anti-MuV-M antibodies.

**Fig 4:**
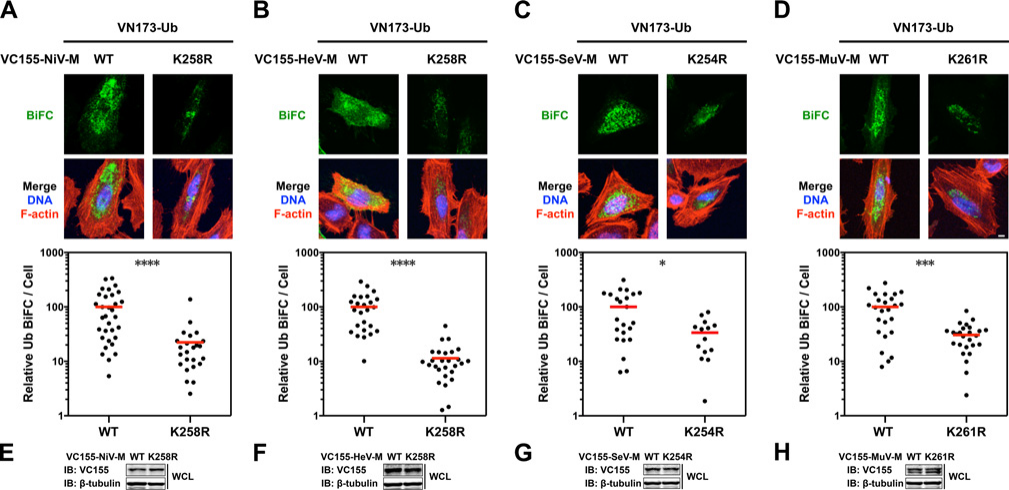
Quantitative BiFC analysis of Nipah, Hendra, Sendai and Mumps virus matrix ubiquitination regulated by a lysine within the NLS_bp_. **(A-D)** Bimolecular fluorescence complementation (BiFC) analysis of ubiquitination of WT or NLS_bp_-lysine mutants of **(A)** NiV-M, **(B)** HeV-M, **(C)** SeV-M and **(D)** MuV-M. Top, Extended Focus (maximum intensity projection) view of 3D confocal micrographs of HeLa cells cotransfected with VN173-Ub and WT, or the indicated NLS_bp_-lysine mutants of VC155-tagged NiV-M, HeV-M, SeV-M or MuV-M. At 24h post-transfection, cells were counterstained with DAPI to visualize nuclear DNA, blue, and fluorescent phalloidin to visualize the F-actin cytoskeleton, red. BiFC fluorescence is pseudocolored green. Scale bar 10 μm. Middle, the background subtracted BiFC fluorescence per cell was quantified from 3D-reconstructed confocal micrographs. *p<0.05; ***p<0.001; ****p<0.0001 by a Mann-Whitney test. **(E-H)** Control immunoblots comparing expression levels of WT or NLS_bp_-lysine mutant of VC155-tagged **(E)** NiV-M, **(F)** HeV-M, **(G)** SeV-M and **(H)** MuV-M in whole cell lysates (WCL) of transfected HeLa cells.

Ubiquitination is a dynamic process determined in part by the rates of conjugation versus de-conjugation, but our co-IP and immunoblot analysis of HA-UbK0-modified 3X-Flag-tagged M is a steady-state assay. It is possible that this assay for ubiquitinated M might not efficiently detect subtle differences that arise from such dynamic processes since i) ubiquitinated 3X-Flag-tagged M proteins are of low stoichiometry relative to unmodified native protein, ii) 3X-Flag-tagged M proteins might be mono- and/or polyubiquitinated on multiple lysines, further obscuring a contribution of any single lysine to the sum total ubiquitination, iii) ubiquitination is reversible, and iv) HA-UbK0 must compete with endogenous ubiquitin. In an attempt to overcome these issues, and to further assess whether NiV-M_K258R_, HeV-M_K258R,_ SeV-M_K254R_ and MuV-M_K261R_ are impaired for ubiquitination, we developed a bimolecular fluorescence conjugation (BiFC) ubiquitination assay in which ubiquitin-conjugation of M produces an irreversible fluorescence signal (S4 Fig.) [63,64]. We fused Ub and M to split N- and C-terminal fragments of the fluorescent protein Venus, VN173 and VC155, respectively. Covalent conjugation of Ub to M brings the spilt Venus fragments into close proximity and allows the two otherwise non-fluorescent Venus fragments to reconstitute a functional fluorophore [65,66]. This complemented Venus will remain associated with M (via VC155-M) even if the VN173-Ub moiety is subsequently cleaved from M by a deubiquitinating enzyme (DUB), preserving an atemporal record of ubiquitin conjugation (S4A Fig.). Analysis of total cellular fluorescence showed that the Ub-M BiFC signal was decreased by almost 80% for the *Henipavirus*-M K258R mutants, confirming the significant role of K258 in ubiquitination of NiV-M (Fig. 3A, Fig. 4A) and HeV-M (Fig. 3B, Fig. 4B). In addition, we determined that the Ub-M BiFC signals for SeV-M_K254R_ (Fig. 4C) and MuV-M_K261R_ (Fig. 4D) were also significantly decreased in BiFC signal by nearly 70% compared to the WT proteins.

### Ubiquitination and a lysine within the NLS_bp_ regulate the subnuclear localization of Nipah, Hendra, Sendai and Mumps virus matrix proteins

**Fig 5:**
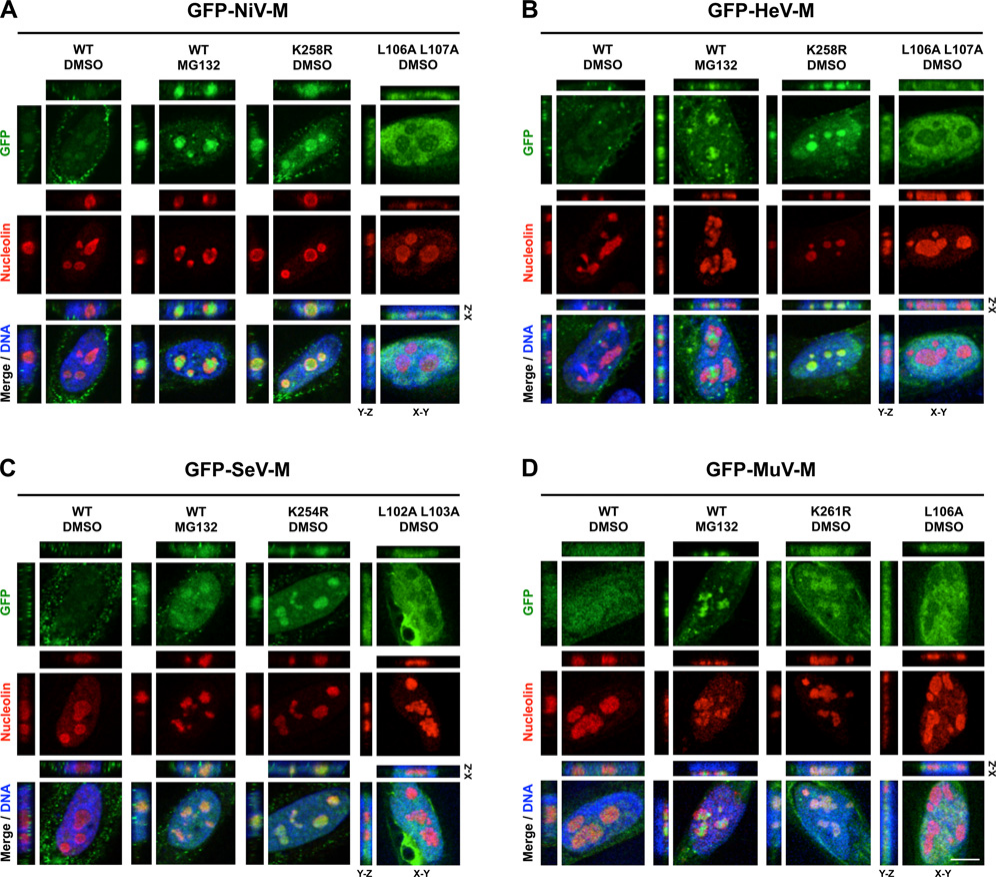
Subnuclear localization of Nipah, Hendra, Sendai and Mumps virus matrix during perturbation of ubiquitination. **(A-D)** XYZ Planes View of 3D confocal micrographs of HeLa cells transfected with WT or the indicated mutants of GFP-tagged **(A)** NiV-M, **(B)** HeV-M, **(C)** SeV-M or **(D)** MuV-M for 16 h then treated with 50 μM MG132/0.5% DMSO or 0.5% DMSO for 8h. Cells were counterstained with DAPI to visualize nuclear DNA, blue, and anti-nucleolin antibodies to visualize nucleoli, red. Scale bar 10 μm.

**Fig 6:**
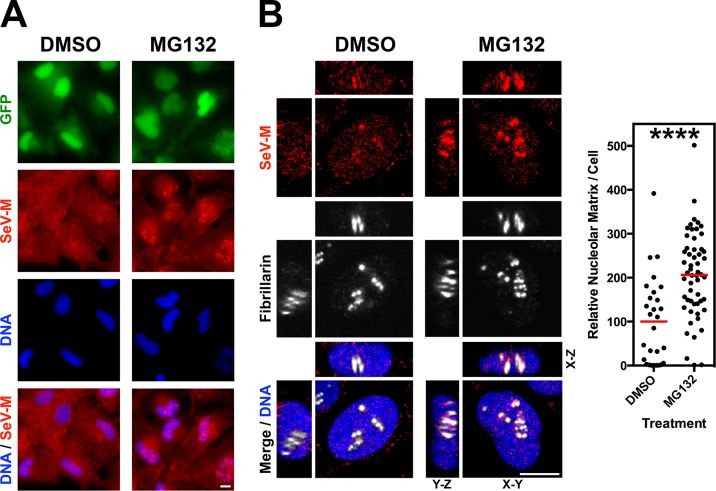
Nucleolar localization of Sendai M during live virus infection and perturbation of ubiquitination. HeLa cells were infected with WT rSeV-eGFP at MOI 10. At 18h post infection, cells were treated with 50 μM MG132/0.5% DMSO or 0.5% DMSO for 8h. **(A)** Wide field epifluorescence of cells counterstained with anti-SeV-M antibodies, red, and with DAPI to visualize nuclei, blue. **(B)** XYZ Planes View of 3D confocal micrographs. Cells were counterstained with DAPI to visualize nuclei, blue, anti-SeV-M antibodies, red, and anti-Fibrillarin antibodies to visualize nucleoli, grayscale. Relative nucleolar localization of SeV-M was determined by its localization to Fibrillarin signal. Scale bars 10 μm. ****p<0.0001 by a Student’s t test.

We observed a punctate localization of GFP-tagged NiV-M, HeV-M, SeV-M, and MuV-M within DNA-void regions of the nucleus when cells were treated with MG132 (Fig. 1B-E). Native untagged NiV-M also exhibited similar subnuclear localization in NiV infected cells treated with bortezomib, an FDA-approved proteasome inhibitor (S1 Fig.). We determined that MG132 redistributes GFP-tagged NiV-M, HeV-M, SeV-M and MuV-M to nucleoli by counterstaining cells with anti-nucleolin antibodies (Fig. 5A-D, second vertical panels). Similarly, treatment of cells with MG132 caused a significant increase in the nucleolar localization of SeV-M during infection with live eGFP-expressing recombinant Sendai virus (rSeV-eGFP). This rSeV-eGFP is derived from a Fushimi strain engineered with mutations that permit replication in mammalian cells without the addition of trypsin as described in Materials and Methods (Fig. 6) [67]. The nucleolar localization of M proteins during ubiquitin depletion predicts that mutations in M that prevent efficient ubiquitination would also cause nucleolar retention. Indeed, GFP-tagged NiV-M_K258R_, HeV-M_K258R_, SeV-M_K254R_ and MuV-M_K261R_ phenocopied the MG132-induced nucleolar localization of the WT proteins (Fig. 5A-D, compare the second and third vertical panels). In contrast, the nuclear localized NES mutants, GFP-tagged NiV-M_L106A 107A_, HeV-M_L106A 107A_, SeV-M_L102A L103A_, and MuV-M_L106A_, were primarily enriched within the nucleoplasm and not the nucleolus. Thus, NES mutants are stalled at a different stage of subnuclear trafficking compared to the NLS_bp_-lysine mutants (Fig. 5A-D, fourth vertical panels). In sum, for the cognate paramyxovirus matrix proteins that exhibit a consistent nuclear trafficking phenotype that is both ubiquitin- and motif-dependent, our data supports a model where proper matrix ubiquitination is required for efficient nucleolar exit and/or preventing retention in the nucleolus.

### Recombinant Sendai virus bearing matrix nuclear export mutants are defective for viral morphogenesis

**Fig 7:**
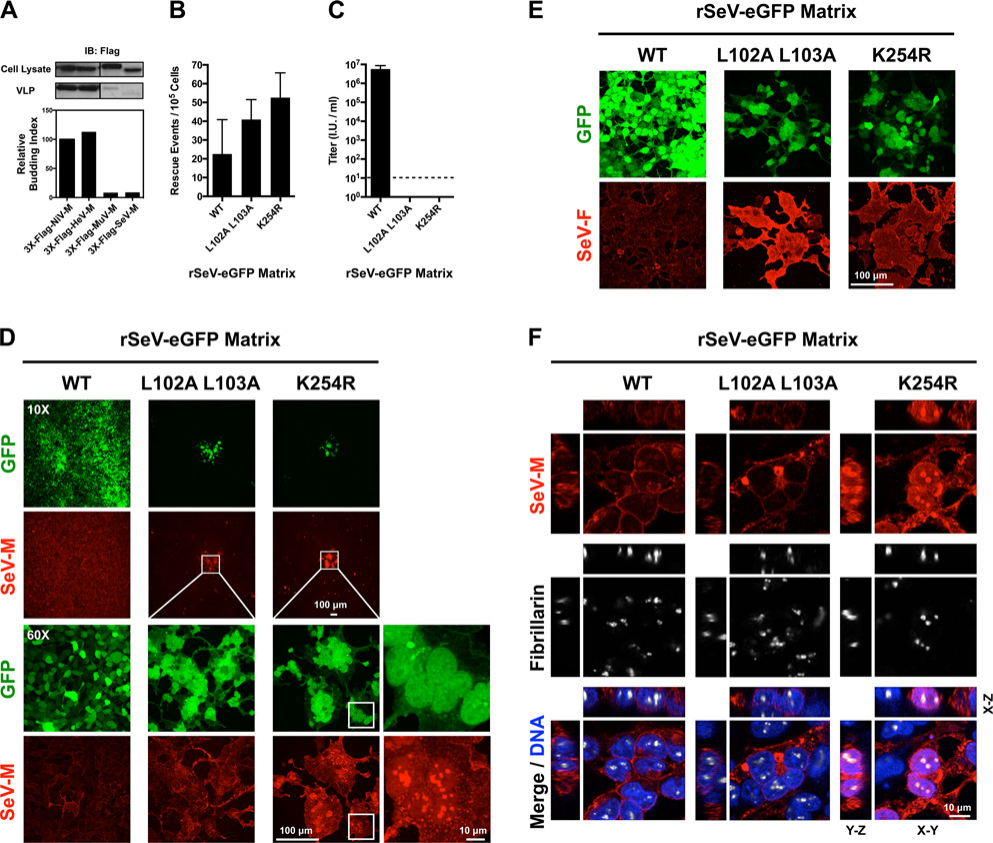
Analysis of rescue efficiency, replication, and matrix localization of recombinant Sendai virus bearing matrix nuclear export mutants. **(A)** Comparison of virus-like particle (VLP) budding between 3X-Flag-tagged NiV-M, HeV-M, MuV-M, and SeV-M. Anti-Flag immunoblots of cell lysate and purified VLPs from HEK 293T cells 24 h post transfection with the indicated constructs. Normalized budding index was calculated from immunoblot integrated intensities. **(B)** Quantification of rescue events (GFP+ cells) from three independent rescues of rSeV-eGFP containing WT, L102 L103 mutant, or K254R mutant SeV-M at day 2 post rescue in HEK 293T cells. **(C)** Quantification of viral titers from the rescues of rSeV-eGFP containing WT, L102 L103 mutant, or K254R mutant SeV-M at day 6 post rescue in HEK 293T cells. **(D)** Extended Focus (maximum intensity projection) view of 3D confocal micrographs of HEK 293T cells at day 6 post rescue of rSeV-eGFP containing WT, L102 L103 mutant, or K254R mutant SeV-M. Cells were counterstained with anti-SeV-M antibodies, red. **(E)** Extended Focus (maximum intensity projection) view of 3D confocal micrographs of HEK 293T cells at day 6 post rescue of rSeV-eGFP containing WT, L102 L103 mutant, or K254R mutant SeV-M. Cells were counterstained with anti-SeV-F antibodies, red. **(F)** XYZ Planes View of 3D confocal micrographs of HEK 293T cells at day 6 post rescue of rSeV-eGFP containing WT, L102 L103 mutant, or K254R mutant SeV-M. Cells were counterstained with DAPI to visualize nuclei, blue, anti-SeV-M antibodies, red, and anti-Fibrillarin antibodies to visualize nucleoli, grayscale.

We previously determined that NiV-M_L106A 107A_ and NiV-M_K258R_ are defective at budding virus like particles (VLPs) [39]. We wanted to compare the effects of the corresponding mutations in 3X-Flag-tagged SeV-M or MuV-M, however these proteins have a poor budding efficiency that is less than 10% of 3X-Flag-tagged *Henipavirus*-M proteins (Fig. 7A). This may be due to the presence of the 3X-Flag-tag or to the fact that SeV-M and MuV-M do not efficiently bud VLPs without the support of other viral proteins [41–43]. To overcome these technical difficulties and to study these mutations in a biologically relevant context, we engineered SeV-M_L102A L103A_ and SeV-M_K254R_ into a recombinant T7-driven, GFP-expressing Sendai virus genome (rSeV-eGFP) that can be rescued as live virus via the cotransfection of support plasmids expressing N, P, L (comprising the necessary replication complex) and a codon-optimized T7 polymerase. This highly efficient reverse genetics system allows us to quantify the number of rescue events directly in transfected producer cells at early time-points (see Materials and Methods). At two days post-transfection, GFP-positive cells (rescue events) could be observed by epifluorescence and quantified by FACS analysis. As a control for background GFP expression in the absence of virus production, we found that cotransfection of WT rSeV-eGFP and T7 polymerase without the N, P and L support plasmids resulted in no GFP-positive cells. We determined that rSeV-eGFP-M_L102A L103A_, and rSeV-eGFP-M_K254R_ rescued at similar if not higher efficiencies than rSeV-eGFP-M_WT_ (Fig. 7B). However, only rSeV-eGFP-M_WT_ produced infectious viral titers (~10^7^ I.U./ml) at day 6 post-rescue, while the mutants did not produce detectible infectious virus (<10 I.U./ml) (Fig. 7C).

To determine the nature of the defect in viral replication, we counterstained the viral rescue cells with anti-SeV-M or anti-SeV-F antibodies and analyzed them by 3D confocal microscopy. By day 6 post-rescue of rSeV-eGFP-M_WT_, infection has spread to all cells without evidence of cell-cell fusion (Fig. 7D). It is known that SeV replication in cell culture does not result in cell-cell fusion [45,47,68], an unusual phenotype as most paramyxovirus infections result in extensive cell-cell fusion (e.g. see S1 Fig. for NiV). Interestingly, although the rSeV-eGFP-M_L102A L103A_ and rSeV-eGFP-M_K254R_ rescue cells did not produce infectious virus, the GFP-positive rescue cells did initiate the formation of large foci of fused cells (Fig. 7D, second and third horizontal panels). Virus-cell and cell-cell fusion require the presence of F and HN [9,10], and we confirmed that SeV-F is expressed on rSeV-eGFP-M_L102A L103A_ and rSeV-eGFP-M_K254R_ foci (Fig. 7E). Recombinant MuV-eGFP genomes engineered with M nuclear export mutants were also unable to efficiently spread beyond the fused cells formed at sites of rescue (S5 Fig.). These data indicate that proper M nuclear-cytoplasmic trafficking is necessary for viral morphogenesis.

To determine the nuclear localization of SeV-M, we counterstained rSeV-eGFP rescue cells with DAPI to visualize nuclei and anti-fibrillarin antibodies to visualize nucleoli. SeV-M_WT_ was primarily extranuclear at the cell periphery (Fig. 7D, 7F). SeV-M_L102A L103A_ did not have an obvious nuclear localization in the viral context, although intracellular inclusions were apparent, suggesting that this mutant nonetheless had an altered localization (Fig. 7F). SeV-M_K254R_, on the other hand, was strongly nuclear and enriched within the nucleoli (Fig. 7D, 7F). These results are consistent with the previous transient transfection experiments in which SeV-M_K254R_ was also more strongly localized to the nucleus than SeV-M_L102A L103A_ (Fig. 7H). Thus, these live virus results support our model that ubiquitination regulates the nuclear and subnuclear trafficking of SeV-M.

### Proteomics analysis supports a model of regulated nuclear transport of *Paramyxovirinae* matrix proteins that involves a critical nucleolar transit phase

Having characterized determinants of *Paramyxovirinae* M nuclear-cytoplasmic trafficking encoded within some M proteins, we turned to identifying potential cellular regulators of this process. We generated inducible 3X-Flag-M-expressing stable HEK 293 cell lines to efficiently copurify M-interacting proteins and analyzed their composition using multidimensional protein identification technology (MudPIT) as described in Materials and Methods (S1–S4 Tables). We opted to determine the protein interactomes of NiV-M, HeV-M, SeV-M and NDV-M since these are the *Paramyxovirinae* M proteins with confirmed nuclear trafficking during live virus infection (Fig. 6, Fig. 7, S10 Fig.) [39,48,51–54,69], and because these proteins cover the widest range of sequence homology to NiV-M: ~90% amino acid identity for HeV-M, ~37% amino acid identity for SeV-M, and ~20% amino acid identity for NDV-M.

S6 Fig. shows our experimental schema and stringent filtering that resulted in our list of putative M protein interactors detailed below and listed in S1–S4 Tables. Nonspecific interactions in MudPIT analyses tend to be independent of the bait of interest. Rather, they are background contaminants related to the cell type and the affinity purification scheme [70]. To remove background contaminants, our putative M interactomes represent only those proteins identified in the sample purifications that are absent in 3 independent negative-control purifications using lysates from the parental/isogenic Flp-In T-REx-293 cells, irrespective of relative abundances (S6 Fig., Worksheet 1 in S1–S4 Tables). As an independent confirmation of stringency, comparison of the putative NiV-M, HeV-M, SeV-M, and NDV-M interactomes to 21 relevant control experiments in the mass spectrometry contaminant repository, CRAPome, revealed relatively few additional proteins that are common sources of contamination (S6 Fig., Worksheet 2 in S1–S4 Tables) [70]. These were primarily actins, tubulins, histones and ribosomal proteins, which are also the most common contaminants across the entire CRAPome (Worksheet 2 in S1–S4 Tables) [70]. During manuscript revisions, another group published the identification of ~130 HeV-M-interacting proteins using affinity-purification, in-gel digestion and mass spectrometric identification, and further characterized AP3B1 as a *Henipavirus* M interactor that regulates VLP production [71]. A majority of their proteins either went undetected by our global analyses or were excluded as background contaminants because they were present in the control purifications. For the purpose of comparison, the proteins in their paper that are also present in our HeV-M and/or NiV-M interactomes, excluding some ribosomal proteins, are KRI1, RFC1, FAM120A, SMC1A, SART3, UPF1, Nat10, Smarca5, UTP14A, POP1, ZC3HAV1, RAD18, AP3D1, USP7, Tat-SF1, SKIV2L2, PARP-1, and Importin-7 (Worksheet 1 in S1 and S2 Tables). Other than PARP-1, these proteins were unlikely to be present as contaminants in the 21 relevant CRAPome control experiments. We noted that the E3 ubiquitin ligase RAD18 was not present as a contaminant in any of the 21 CRAPome control experiments and was the least likely to be encountered in the entire CRAPome database; it was found in only 4 of 411 experiments with an average of only 1.3 spectra per experiment, whereas we measured 11 unique spectra (22.6% coverage) in the NiV-M affinity purification [70]. We confirmed the interaction of NiV-M with RAD18 by co-IP and immunoblot analysis (S7A Fig.), indicating that a combination of experimental and computational approaches to background contaminant subtraction can facilitate the identification and characterization of bona fide protein-protein interactions (S6 Fig.). Other ubiquitin ligases identified in our proteomics experiments (UBE2O and Cullin ring ligases) were also confirmed for copurification with M proteins by co-IP and immunoblot analysis (S7B–C Fig.).

**Fig 8:**
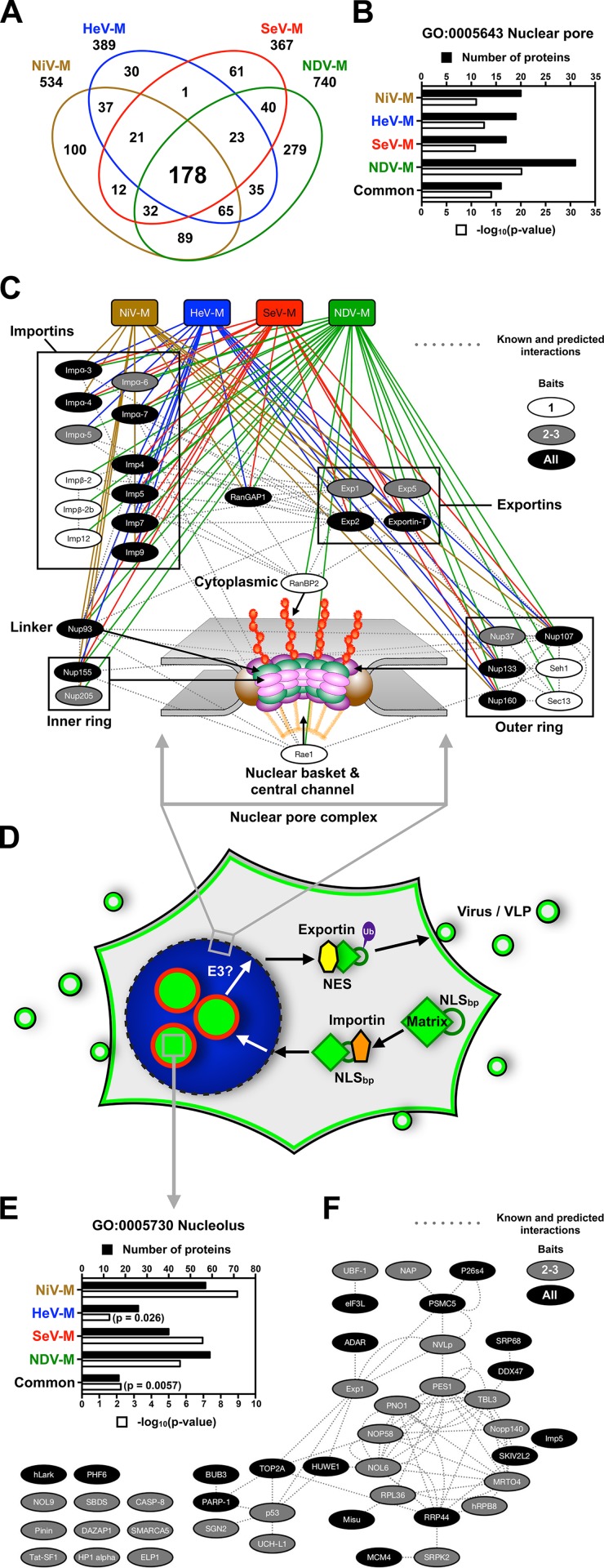
Identification of nuclear pore complex proteins, nuclear transport receptors and nucleolar proteins that interact with *Paramyxovirinae* matrix proteins. **(A)** Overlap of NiV-M, HeV-M, SeV-M and NDV-M protein interactomes identified by MudPIT analysis. **(B)** Functional annotation enrichment of proteins associated with the nuclear pore within the NiV-M, HeV-M, SeV-M, NDV-M and common interactomes using DAVID Bioinformatics Resources. **(C)** Protein-protein interaction network of nuclear pore complex proteins and nuclear transport receptors within the NiV-M, HeV-M, SeV-M and NDV-M interactomes using Cytoscape with the GeneMANIA plugin. **(D)** A model for matrix nuclear-cytoplasmic trafficking. Import through the nuclear pore complex is mediated by the interaction between an importin and the NLS_bp_ of M proteins. Nuclear export is mediated by the interaction between an exportin and the NES of M proteins. Ubiquitination of some matrix proteins by as yet unknown E3 ubiquitin-ligases regulates nuclear export and/or nuclear re-import by masking the NLS_bp_. **(E)** Functional annotation enrichment of proteins associated with the nucleolus within the NiV-M, HeV-M, SeV-M, NDV-M and common interactomes using DAVID Bioinformatics Resources. **(F)** Protein-protein interaction network of a subset of proteins associated with the nucleolus within the NiV-M, HeV-M, SeV-M and NDV-M interactomes using Cytoscape with the GeneMANIA plugin. M proteins and the nucleolar proteins bound by only one M-bait were omitted for clarity.

Confident that our putative M interactomes were largely reflective of true protein-protein interactions, we further analyzed the interactomes bioinformatically and biochemically (Fig. 8, S7–S9 Fig.). Comparisons of our putative NiV-M, HeV-M, SeV-M, and NDV-M interactomes to one another revealed significant overlap; over 60% of the proteins found in any single interactome were also found in the interactomes of one or more of the other three (Fig. 8A). Furthermore, we identified 178 proteins common to all M interactomes, the majority of which are not present in the 21 historical control experiments (Worksheet 3 in S4 Table). This common set of proteins represents 24–48% of all the proteins in any single viral M interactome (Fig. 8A, S1–S4 Tables). Interestingly, proteins associated with the nuclear pore complex were significantly enriched within individual M interactomes as well as the subset of common interacting proteins (Fig. 8B,-log_10_(p-value)>10). These include nuclear pore complex components (RanBP2, Nup37, Nup93, Nup107, Nup155, Nup205, Sec13, Seh1), nuclear transport receptors (NTRs) required for nuclear import of proteins (α/β-importins), nuclear export of proteins (Exp1/CRM1, Exp2), nuclear export of dsRNA/dsRNA-binding proteins (Exp5), nuclear export of tRNA (Exportin-T), nuclear export of mRNA (Rae1), and a regulator of the RanGTP/GDP cycle that modulates the association/dissociation of cargo with NTRs (RanGAP1) (Fig. 8C, Worksheet 1 in S1–S4 Tables). All of these proteins were unlikely background contaminants (Worksheet 2 in S1–S4 Tables) and we confirmed that NiV-M interacts with α-importins and Exp1/CRM1 (S8 Fig.). Thus *Paramyxovirinae* M proteins interact with a highly interconnected network of proteins necessary for transport of NLS and NES containing cargo proteins across the nuclear pore.

Our cell biological and proteomic findings are synthesized into a working model for ubiquitin-regulated nuclear-cytoplasmic trafficking of M proteins shown in Fig. 8D. Consistent with the nucleolar transit phase exhibited by the *Paramyxovirinae* M proteins under study, the M interactomes also revealed a significant enrichment of resident or transient nucleolar proteins (Fig. 8E and 8F). One of these, the RNA polymerase I transcription factor UBF-1 (UBTF) had abundant spectral counts in the SeV-M and NiV-M interactomes but was essentially absent in the matched CRAPome control experiments (S1 Table and S3 Table). We determined that UBF-1 sequesters NiV-M in the nucleus and inhibits NiV-M budding when overexpressed (S9 Fig.). Thus, our proteomic and functional data indicate that M proteins interact with an array of nuclear and nucleolar proteins, at least some of which can modulate M nuclear-cytoplasmic trafficking.

## Discussion

Whether or not they replicate in the nucleus, many viruses are known to target, modify, and hijack nuclear components and nuclear functions to promote the infectious life cycle. It is generally thought that paramyxoviruses replicate in the cytosol without a nuclear stage. However, it is becoming increasingly clear that nuclear trafficking of M is shared by a number of paramyxoviruses. It was previously observed that SeV-M, NDV-M and RSV-M traffic through the nucleus [49,50,69] and a functional bipartite nuclear localization signal (NLS_bp_) has been defined within NDV-M [48]. Here, we show that the NLS_bp_ of NDV-M is functionally conserved for nuclear import along with NiV-M, HeV-M, SeV-M and MuV-M (Fig. 8D, S3 Fig.) [39,48]. NLSs specify translocation through the nuclear pore through high-affinity interactions with importins, which in turn interact with cognate nuclear pore components on the cytoplasmic side [72]. In our proteomic analyses we identified numerous importins, exportins and nuclear pore complex components as common candidate interactors of NiV-M, HeV-M, SeV-M and NDV-M (Fig. 8, S8 Fig., S1–S4 Tables). Thus, the size of *Paramyxovirinae* M proteins (>40 kDa), the presence of a functional NLS_bp_ within M proteins, and the interaction with nuclear transport receptors are strong evidence that the nuclear localization of M proteins is an active and regulated transport process. That the putative M interactomes show such a strong enrichment of proteins involved in nuclear-cytoplasmic transport also suggests that M proteins may antagonize the nuclear-cytoplasmic trafficking of host proteins and RNA to facilitate viral replication [73].

In addition to the NLS_bp_, we show that a leucine-rich NES sequence is functionally conserved within NiV-M, HeV-M, SeV-M and MuV-M (Fig. 2, Fig. 5, Fig. 8D). We note that mutation of the corresponding region in NDV-M resulted in decreased nuclear localization. However, this sequence is not as well conserved as in the other M proteins (Fig. 2A) and a recent study identified other functional NES motifs within different regions of NDV-M [52]. Thus, *Paramyxovirinae* M proteins appear to have both shared and unique determinants of nuclear-cytoplasmic trafficking depending on their evolutionary heritage. We also acknowledge that our experimental system utilizing human cells may not fully recapitulate the regulation of NDV-M trafficking since NDV is an avian virus, while NiV, HeV, SeV, MuV and MeV are mammalian viruses.

Nuclear export of NiV-M, HeV-M, SeV-M and MuV-M is also regulated by a lysine within the second basic patch of the NLS_bp_. Mutating this lysine to an arginine results in decreased ubiquitination and a nuclear retention phenotype that is phenocopied by pharmacological depletion of free ubiquitin with a proteasome inhibitor (Fig. 1B-E, Fig. 2B-E, Fig. 5, Fig. 6). We hypothesize that mutation of this lysine prevents its ubiquitination. Alternately, mutation of this lysine may prevent the ubiquitination of a nearby lysine within the NLS_bp_ by preventing the interaction of M with a ubiquitin ligase. Whether ubiquitination regulates MeV-M or NDV-M function(s) remains indeterminate. The subcellular localization phenotypes of the NES and the various NLS_bp_ mutants for NDV-M and MeV-M are not congruent (compare S3 Fig. and Fig. 2F-G). Furthermore, NDV-M and MeV-M also exhibit divergent sensitivity with regards to ubiquitin conjugation (S2E–F Fig.), and neither were found to be sensitive to MG132 induced nuclear retention (Fig. 1F-G). Altogether, our results suggest that the degree of the nuclear trafficking phenotypes of transfected M proteins mutated at the putative NES, the putative NLS_bp_, or the homologously aligned lysine within the NLS_bp_, are strongest for the *Henipavirus* M proteins, moderate for SeV-M and MuV-M, variable for NDV-M, and inconclusive/absent for MeV-M under the cell type and conditions examined (Table 1). Although we did not find conclusive evidence for ubiquitination of NDV-M on the lysine that corresponds to the *Henipavirus* M K258, it is probable that NDV-M is a biological target of posttranslational modification on other lysines within the NLS_bp_. Our proteomic analysis detected a peptide from the NLS_bp_ of NDV-M containing 114.0492 Da mass signatures indicative of the vestigial diglycine of ubiquitin that remains attached to the modified lysine (**K**[114.04292]) after trypsin cleavage (^249^GK**K**[114.04292]VTFD**K**[114.04292]LEKKIRSLDLSVGLSDVLGPSVLVK^281^) [74].

How could NLS ubiquitination regulate nuclear trafficking? Protein import into the nucleus is regulated by the affinity of importins for cargo NLSs, which can be modulated by intermolecular or intramolecular masking of the NLS itself [75,76]. For example, ubiquitination of the NLS of p53 by MDM2 has been shown to block p53 nuclear import by preventing the binding of importin-α3 [77]. It was recently shown that the ubiquitin-conjugating enzyme UBE2O multi-monoubiquitinates tumor suppressor BAP1 on its NLS_bp_ to promote cytoplasmic localization. It was further determined that UBE2O specifically binds and ubiquitinates a number of similar bipartite NLSs within nuclear trafficking proteins known to regulate RNA processing, transcription, DNA replication, and chromatin remodeling [78]. We hypothesize that ubiquitination of M on a lysine within the NLS_bp_ itself prevents importin binding as a means to prevent nuclear re-entry once the protein has completed its nuclear sojourn (Fig. 8D, S8A Fig.). Given this model for ubiquitin-dependent nuclear-cytoplasmic trafficking, it is possible that M utilizes a nuclear-resident E3 ubiquitin ligase [79]. Our proteomic analyses identified a number of candidate ubiquitin ligases that interact with M proteins including UBE2O, which was found within the NiV-M and NDV-M interactomes (S7B Fig., S1 Table, S4 Table). Further study of these ubiquitin ligases will help resolve the spatiotemporal dynamics of M ubiquitination vis-à-vis nuclear trafficking (S7 Fig.).

Nuclear-cytoplasmic trafficking is a prerequisite for M budding and viral morphogenesis. We previously showed that NiV-M mutants defective in either nuclear import or nuclear export were also defective at budding VLPs [39]. Alternately, overexpression of a nuclear NiV-M-interacting protein UBF-1 can sequester NiV-M in the nucleus and inhibits efficient budding of VLPs (S9 Fig.). Other viruses have been reported to interact with UBF-1 and utilize or modify UBF-1 function. For example, UBF-1 is inactivated during Poliovirus infection as part of a viral strategy to inhibit host cell transcription globally [80]. The DNA viruses Adenovirus and HSV-1 co-opt UBF-1 into viral DNA replication centers, and it has been hypothesized that UBF-1 is used as a cofactor in viral DNA replication [81–83]. Finally, the SV40 large T antigen and the HCV NS5A protein stimulate RNA Pol I transcriptional activity and enhance rRNA synthesis by hyperphosphorylation of UBF-1 [84,85]. Such rRNA transcriptional activation is thought to contribute to the cell transformation caused by these tumorigenic viruses. For NiV, even though NiV-M clearly interacts with overexpressed UBF-1, and is colocalized with UBF-1 in the nucleus, it is unclear whether NiV utilizes UBF-1 to benefit viral replication under physiological expression levels and conditions (S9 Fig.). Nonetheless, the inhibitory effects of nuclear UBF-1 on VLP budding supports the model that functional trafficking to the plasma membrane requires properly regulated nuclear import and export (Fig. 8D).

Here, we also engineered M nuclear export mutants into recombinant SeV. rSeV-eGFP-M_L102A L103A_ or rSeV-eGFP-M_K254R_ were completely attenuated for production of infectious virus, but formed large foci of fused cells at sites of viral rescue. Moreover, nuclear localization of SeV-M_K254R_, the ubiquitination mutant, was observed in both virus rescue and transient transfection experiments (Fig. 2D, Fig. 5C, Fig. 7F). It is known that mutations that abrogate the interaction of M with the glycoproteins, including M deletion, can increase cell-cell fusion in SeV and MeV, while mutations that enhance their interaction can decrease cell-cell fusion [33,34,45–47,68]. Since M and F proteins were expressed in the foci of fused cells, our results indicate that rSeV-eGFP-M_L102A L103A_ and rSeV-eGFP-M_K254R_ are defective in proper assembly of viral components at the plasma membrane rather than in expression of viral components necessary for budding *per se*. The link between viral replication and M ubiquitin-dependent nuclear-cytoplasmic trafficking may explain why proteasome inhibitors that deplete free cellular pools of ubiquitin have been found to inhibit SeV and NiV replication [39,86].

Beyond regulating nuclear import/export itself, we previously found that ubiquitination of NiV-M is necessary for membrane targeting and budding [39]. It is possible that the ubiquitination of M proteins promotes recognition by cellular factors such as ESCRT complexes known to mediate transport and budding of many enveloped viruses [56,87,88], especially in light of known sequence motifs in PIV5-M, SeV-M and MuV-M that can bind ESCRT complex components [27,43,89]. The status of M ubiquitination may also regulate the interactions of M with cellular factors inside the nucleus and within subnuclear compartments such as the nucleolus. A number of cellular proteins become enriched in the nucleolus upon proteasome inhibition, including p53 [90–99]. Similarly, pharmacological or genetic inhibition of NiV-M, HeV-M, SeV-M, and MuV-M ubiquitination sequesters these proteins in the nucleolus (Fig. 5, Fig. 6), and nucleolar localization of SeV-M_K254R_ was also confirmed in the context of rSeV-eGFP rescue (Fig. 7F). Nucleolar localization of M proteins is a natural feature of the nuclear sojourn of some M proteins, with M enriched at nucleoli during the early stage of live NiV and NDV infections (S10 Fig.) [69,100]. Although M proteins do not have an evident nucleolar localization signal (NoLS) that would have predicted this observation [101], our proteomics experiments suggest that *Paramyxovirinae* M proteins can interact with a number of nucleolar hub proteins (Fig. 8E and F, S1–S4 Tables) [102].

Most, if not all, viral families interact with the nucleolus, often to usurp cellular functions and promote viral replication [103–105], as the nucleolus is a dynamic structure involved in a vast array of biological functions beyond ribosome biogenesis, including tRNA and mRNA processing and export from the nucleus, cell cycle regulation, and response to cellular stress. Additionally, there is growing recognition that NLS containing viral proteins target the nuclear pore complex to alter the export of macromolecules and mRNA, thereby counteracting antiviral responses and promoting viral gene expression at the expense of host gene expression [73]. For example, influenza NS1 is a multifunctional protein known to translocate to the nucleolus and to the nuclear pore where it inhibits host mRNA export factors resulting in impaired immune responses and enhanced viral virulence [106]. Vesicular stomatitis virus M also inhibits mRNA nuclear export through interaction with nuclear pore components [73,107]. Further, RSV-M is shuttled to the host cell nucleus where it inhibits host gene expression and induces cell cycle arrest, indicating that paramyxovirus M proteins also antagonize nuclear functions [108,109]. We hypothesize that ubiquitin-dependent nuclear and subnuclear trafficking of some *Paramyxovirinae* M proteins is part of a viral strategy to promote viral replication. Therefore, the study of M interactions with the nucleolus and the nuclear pore complex represents an opportunity to gain new insights into the cell biology of the nucleus and to identify novel antiviral targets.

## Materials and Methods

### Cell culture and transfection

HeLa, Vero, and HEK 293T cells were maintained at 37°C in a 5% CO_2_ atmosphere in Dulbecco’s modified Eagle’s medium (DMEM) supplemented with 10% fetal bovine serum (FBS) and 1% 100X penicillin/streptomycin solution (Gibco/Life Technologies, Gaithersburg, MD). For confocal microscopy imaging, cells were seeded on 22 mm #1.5 coverglass coated with Collagen Type I (BD Biosciences, San Jose, California). Cells were transfected using Lipofectamine LTX per the manufacturer’s instructions (Invitrogen/Life Technologies). 3X-Flag-M Flp-In T-REx-293 cell lines, generated as described below, were maintained at 37°C in a 5% CO_2_ atmosphere in DMEM supplemented with 10% dialyzed FBS and 1% 100X penicillin/streptomycin solution. 3X-Flag-M protein expression was induced and immunoprecipitated as described below.

### Plasmids, cell lines and virus reverse genetics constructs

3X-Myc-tagged Cullin constructs (Addgene plasmids 19896, 19892, 19893, 19951, 19922, 19895 and 20695) are described in [110–113]. GFP-UBF-1 (Addgene plasmid 17656) is described in [114]. Flag-tagged Karyopherin constructs were the kind gift of Dr. Christopher F. Basler (Icahn School of Medicine at Mount Sinai, New York, NY). 3XFlag-CRM1 (Addgene plasmid 17647) is described in [115]. The Myc-UBE2O construct was the kind gift of Dr. El Bachir Affar (Maisonneuve-Rosemont Hospital Research Center, Department of Medicine, University of Montreal, Montreal) and is described in [78]. HA-UbK0 (Addgene; plasmid 17603; all lysines mutated to arginines) is described in [55]. Codon optimization and cloning of untagged, 3X-Flag-tagged and 3X-Flag-GFP-tagged Nipah virus matrix (NiV-M) and generation of corresponding NiV-M mutants is described in [39]. We similarly codon optimized and cloned the open reading frames encoding M from Hendra virus (HeV-M, genus *Henipavirus*), Sendai virus (SeV-M, genus *Respirovirus*), Mumps virus (MuV-M, genus *Rubulavirus*), and Measles virus (MeV-M, genus *Morbillivirus*), and also a non-codon optimized Newcastle disease virus M (NDV-M, genus *Avulavirus*): briefly, eGFP was fused to the N-terminus of M by overlap extension PCR (OE-PCR). WT or GFP-fused HeV-M, SeV-M, RSV-M, MuV-M, and MeV-M were inserted within the HindIII and XhoI sites of pCMV-3Tag-1, while NDV-M was inserted within HindIII and ApaI sites of pCMV-3Tag-1 (Agilent Technologies, Santa Clara CA) to generate 3X-Flag- and 3X-Flag-GFP-tagged-M constructs. Alignment of M sequences using Clustal Omega identified sequences motifs corresponding to NiV-M’s nuclear export sequence (NES) and bipartite nuclear localization sequence (NLS_bp_) [39,116]. Mutations were generated using the QuikChange II site-directed mutagenesis kit using PAGE-purified mutagenesis primers designed using the online QuikChange primer design tool (Agilent Technologies). A RAD18 cDNA clone was purchased from Origene (SC323786). 3X-Flag-GFP-RAD18 was generated by replacement of the NiV-M insert in HindIII/XhoI digested 3X-Flag-NiV-M.

The Flp-In T-REx system (Invitrogen) was used to generate doxycycline-inducible 3X-Flag-M cell lines. Codon-optimized 3X-Flag-tagged NiV-M, HeV-M, SeV-M, and NDV-M were inserted within the KpnI and XhoI sites of pcDNA5/FRT/TO. The constructs and pOG44 were cotransfected into Flp-In T-REx-293 cells, and stable cell lines were selected with hygromycin and blasticidin according to the manufacturer’s instructions.

Constructs for bimolecular fluorescence complementation (BiFC) analyses were generated with split Venus residues 1–172 (VN173) and 155–238, A206K (VC155) [65,117]. VN173 and VC155 were PCR amplified from pBiFC-VN173 (Addgene plasmid 22010) and pBiFC-VC155 (Addgene plasmid 22011), and were fused to the N-termini of Ub and M proteins via a flexible linker encoding GGGGSGGGGGR by OE-PCR. VN173-Ub and VC155-M were inserted within the NotI and XhoI sites of pcDNA3.1(+) (Life Technologies). Mutations within VC155-M constructs were generated using the QuikChange II site-directed mutagenesis kit using PAGE-purified mutagenesis primers designed using the online QuikChange primer design tool (Agilent Technologies).

The recombinant Sendai virus (rSeV) anti-genome RGV0, a Fushimi strain construct with F1-R strain mutations in F and M, and helper plasmids encoding SeV N, P and L were the kind gift of Dr. Nancy McQueen and are described in [67]. The encoded virus has the ability to replicate in mammalian cells without the addition of trypsin. We further modified the rSeV anti-genome construct by inserting an eGFP reporter flanked at the 3’ end by a unique NotI site between the N and P genes. A hammerhead ribozyme sequence was inserted between the optimal T7 promoter and the start of the anti-genome. Mutations were introduced into the SeV-M ORF by OE-PCR using primers containing the desired mutations, followed by insertion into NotI and AfeI sites in the parental rSeV-eGFP construct.

The full-length construct encoding recombinant Mumps virus (rMuV) anti-genome of the Jeryl Lynn 5 (JL5) vaccine strain and helper plasmids encoding MuV-JL5 N, P and L proteins were a kind gift from Dr. W. Paul Duprex and are described in [118]. We modified the rMuV anti-genome construct by inserting an eGFP reporter between the NP and P genes. A hammerhead ribozyme sequence was inserted between the optimal T7 promoter and the start of the anti-genome. Mutations were introduced into the MuV-M ORF by OE-PCR using primers containing the desired mutations, followed by insertion into SalI and SbfI sites in the parental rMuV construct.

### Multidimensional protein identification technology (MudPIT) analysis of matrix interactomes

3X-Flag-M Flp-In T-REx-293 cell lines were grown to ~80% confluency and induced for protein expression with 100 ng/mL doxycycline for 24h. Cells were washed three times in dPBS and lysed in 100 mM Tris-HCL pH 8, 150 mM NaCL, 5 mM EDTA, 5% glycerol, 0.1% NP40, complete protease cocktail (Roche), PhosSTOP (Roche) and 25 mM N-ethylmaleimide. Cell lysate was clarified by centrifugation at >15,000×g for 15 min at 4°C and incubated with lysis buffer-equilibrated anti-Flag M2 affinity gel (Sigma-Aldrich, St. Louis, MO) for 2 hours at 4°C. The affinity gel was extensively washed with lysis buffer and then with elution buffer consisting of 100 mM Tris-HCL pH 8, 150 mM NaCL, 5 mM EDTA, and 5% glycerol. Bound proteins were eluted from the affinity gel with elution buffer containing 3X-Flag peptide (Sigma-Aldrich), were precipitated with trichloroacetic acid, washed with acetone twice, dried, and stored at -20°C until further processing.

Protein samples were resuspended in 8M urea in 100 mM Tris pH 8.5, reduced, alkylated and digested by the sequential addition of lys-C and trypsin proteases as previously described [119]. The digested peptide solution was fractionated online using strong-cation exchange and reverse phase chromatography and eluted directly into an LTQ-Orbitrap mass spectrometer (Thermofisher) [119,120]. MS/MS spectra were collected and subsequently analyzed using the ProLuCID and DTASelect algorithms [121,122]. Database searches were performed against a human database containing the relevant paramyxovirus M protein sequence. Protein and peptide identifications were further filtered with a false positive rate of less than 5% as estimated by a decoy database strategy [123]. Normalized spectral abundance factor (NSAF) values were calculated as described [124]. Proteins were considered candidate M-interacting proteins if they were identified in the relevant affinity purification but not present in 3 independent control purifications using lysates from the parental Flp-In T-REx-293 cells. Analysis of other potential background contaminants was performed using CRAPome [70]. Venn diagrams were generated using jvenn [125]. Gene-annotation enrichment analysis was performed using DAVID Bioinformatics Resources 6.7 [126,127]. Physical and predicted protein interaction networks were visualized using the GeneMANIA plugin for Cytoscape 3.1 [128,129].

### Immunoprecipitations and immunoblot analysis of matrix ubiquitination

Transfected HEK 293T cells were washed once in dPBS and lysed in 100 mM Tris-HCL pH 8, 150 mM NaCL, 5 mM EDTA, 5% glycerol, 0.1% NP40, complete protease cocktail (Roche) and 25 mM N-ethylmaleimide. The cell extract was clarified by centrifugation at >15,000×g for 15 min at 4°C before incubation overnight at 4°C with lysis buffer-equilibrated anti-Flag M2 affinity gel (Sigma-Aldrich, St. Louis, MO). The affinity gel was extensively washed with lysis buffer and then with elution buffer consisting of 100 mM Tris-HCL pH 8, 150 mM NaCL, 5 mM EDTA, and 5% glycerol. Bound proteins were eluted from the affinity gel with elution buffer containing 3X-Flag peptide (Sigma-Aldrich), were subjected to SDS-PAGE and transferred to immobilon-FL PVDF membrane (EMD Millipore, Billerica, MA). To analyze M ubiquitination by immunoblot, HEK 293T cells were cotransfected with 3X-Flag-M and HA-UbK0 for 24h and subjected to immunoprecipitation as described above. Membranes were simultaneously probed with mouse anti-Flag M2 primary antibodies (Sigma-Aldrich) and rabbit anti-HA primary antibodies (Novus Biologicals, Littleton, CO) followed by anti-mouse-680 and anti-rabbit-800 secondary antibodies (LI-COR, Lincoln, Nebraska) and imaged on an Odyssey infrared scanner (LI-COR) according to the manufacturer’s instructions. To quantify relative ubiquitination, the background subtracted integrated fluorescence intensities of the monoubiquitin bands (Ub) normalized to total M (M_0_+M_1_) was determined using LI-COR Odyssey software.

### Depletion of cellular free ubiquitin

For 3D confocal microscopy analysis, transfected HeLa cells were treated with 50 μM MG132 or 0.5% DMSO at 16 h post-transfection for 8 hours, then fixed and processed for quantitative image analysis as described below. For immunoblot analysis of M ubiquitination during ubiquitin depletion, transfected HEK 293T cells were treated with 10 μM MG132 or 0.1% DMSO at 18 h post-transfection for 6 hours, and 3X-Flag-M was immunoprecipitated as described above.

### Quantification of virus-like particle budding

VLP budding assays were performed as described in [39]. Briefly, precleared supernatants from 3X-Flag-M transfected HEK 293T were ultracentrifuged through a 20% (w/v) sucrose at 36,000 rpm for 2 h at 4°C (AH-650 rotor, Thermo Scientific). VLP pellets and cells were resuspended in lysis buffer and subjected to SDS-PAGE and anti-Flag immunoblotting. Relative integrated intensity of VLP/cell lysate bands were quantified and normalized relative to the budding of 3X-Flag-NiV-M.

### Nipah virus infection and recombinant virus rescue

HeLa cells were infected with Nipah virus under biosafety level 4 (BSL-4) conditions as described in [39]. For rescue of WT or mutant rSeV-eGFP, 2X10^6^ HEK 293T cells were transfected with recombinant plasmid encoding the anti-genome (4 μg) along with the cognate accessory plasmids encoding SeV NP (1.44 μg), P (0.77 μg), and L (0.07 μg), and a codon optimized T7 RNA polymerase (4 μg) using Lipofectamine LTX (8.9 μL) and Plus Reagent (5.5 μL), according to manufacturer’s instructions. Cells were harvested for FACS analysis at 48 hours post-transfection (the earliest time point when GFP-positive cells can be observed by epifluorescence microscopy, yet when supernatant titer is still not detectable) to quantify rescue efficiency. The number of GFP-positive cells (rescue events) was determined from 500,000 cells analyzed with a FACSCalibur Flow Cytometer (BD Biosciences) and FlowJo software (TreeStar Inc., Ashland, OR). Cells plated on coverslips were fixed at day 6 post-transfection for analysis of rescued virus infection by 3D confocal microscopy. Supernatant was collected from rescue cells at day 6 post-transfection for quantification of viral titers. Briefly, supernatant stored at -80°C was thawed on ice and serial diluted 2-fold in serum-free DMEM. 100 μL of each dilution was used to infect ~60,000 Vero cells in a 24-well plate for 1 hour. After 1 h, 500 μL of DMEM 10% FBS was added to each well and the cells were incubated at 37°C. Cells were harvested for FACS analysis at 24 h post infection and titers were calculated based on percent infection in the linear range of supernatant dilutions. For rescue of WT or mutant rMuV, 4X10^5^ BSR-T7 cells were transfected with recombinant plasmid encoding the anti-genome (5 μg) along with the cognate accessory plasmids encoding MuV NP (0.3 μg), P (0.1 μg), and L (0.2 μg), and a codon optimized T7 RNA polymerase (2 μg) using Lipofectamine LTX (18.75 μL) and Plus Reagent (7.5 μL), according to manufacturer’s instructions.

### Microscopy and antibodies

Nipah virus-infected cells were fixed in 10% formalin solution for a minimum of 24 h prior to removal from the BSL-4 laboratory. For all other immunofluorescence microscopy, samples were fixed with 2% paraformaldehyde in 100 mM phosphate buffer (pH 7.4) for 15 min. Fixed cells were permeabilized in blocking buffer containing PBS, 1% saponin, 3% bovine serum albumin, and 0.02% sodium azide. After incubation with antibodies/probes in blocking buffer, samples were extensively washed in blocking buffer and mounted on glass slides with Vectashield mounting medium with DAPI (Vector Laboratories, Burlingame, California, United States). The samples were imaged with a Leica SP5 confocal microscope (Leica Microsystems, Buffalo Grove, IL), acquiring optical Z-stacks of 0.3–0.5 μm steps. Z-stacks were reconstructed and analyzed in three dimensions using Volocity 5.5 software (Perkin Elmer, Waltham, Massachusetts). Widefield microscopy was performed using a Cytation 3 Cell Imaging Multi-Mode Reader (BioTek, Winooski, VT) or a Nikon TE300 microscope. NiV-M was detected with rabbit anti-NiV-M antibodies (1:1000) [39]. SeV-M was detected with mouse anti-SeV-M ascites (1:200), and SeV-F was detected with mouse anti-SeV-F ascites (1:200) kindly provided by Dr. Toru Takimoto [130]. Nucleoli were detected with mouse anti-nucleolin antibodies (1:500) (Invitrogen/Life Technologies) or rabbit anti-fibrillarin antibodies (1:500) (Abcam, Cambridge, MA). Alexa-fluor conjugated Anti-IgG antibodies of appropriate species reactivity and fluorescence spectra were used for secondary detection (1:300–1:1000) (Invitrogen/Life Technologies). F-actin was visualized by incubating samples with Alexa-fluor conjugated phalloidins (1:300) (Invitrogen/Life Technologies).

Immunoblots were imaged on an Odyssey infrared scanner (LI-COR) using secondary antibodies of appropriate species reactivity and fluorescence spectra (LI-COR). An antibody to the C-terminus of GFP (LS-C51736, LifeSpan BioSciences, Inc., Seattle, WA) was used for immunoblot detection of VC155-fusion proteins. Anti-Flag M2 (F3165, Sigma-Aldrich) was used to for immunoblot detection of Flag-tagged proteins. Anti-HA (NB600–363, Novus Biologicals, Littleton, CO) was used for immunoblot detection HA-tagged proteins. Anti-Myc Tag, clone 4A6 (05–724, Millipore, Temecula, CA) was used for immunoblot detection of Myc-tagged proteins. Anti-UBE2O (NBP1–03336, Novus Biologicals) was used for immunoblot detection of UBE2O. Anti-β-Tubulin (T7816, Sigma-Aldrich) and Anti-COX IV (926–42214, Licor) were used for loading controls.

### Quantitative analysis of 3D confocal micrographs

Random 40X fields were imaged using acquisition settings ensuring no under-saturated or over-saturated pixel intensities. Volocity 5.5 software was used for quantitative analysis of 3D confocal images. To determine the quantity of nuclear M, the nuclear compartment was defined with the find objects function within the DAPI-fluorescence channel. Holes in objects (DNA-absent regions such as nucleoli) were filled, and fluorescent objects smaller than nuclei were excluded. The entire cell body was defined by drawing a region of interest (ROI) encompassing all F-actin staining. The sum of voxel intensities in the GFP channel was measured within these defined sets. The average voxel fluorescence of untransfected cells was used for background subtraction. To determine the quantity of nucleolar M, the nucleolus was defined with the find objects function within the Fibrillarin-stained-fluorescence channel. Nucleolar objects were grouped within their respective nuclei, defined as above based on the DAPI-fluorescence channel. To quantify fluorescence from bimolecular fluorescence complementation images, a ROI was drawn around cells fluorescent in the YFP channel. The average voxel fluorescence of untransfected cells was used for background subtraction.

### Statistical analysis

For analysis of M nuclear localization, p-values were generated with a Student’s t test when analyzing two sample groups. To analyze three or more sample groups, p-values were generated by ANOVA with Bonferroni correction for multiple comparisons. To analyze BiFC experiments, p-values were generated using a Mann-Whitney test. All graphs and statistical analyses were generated with Prism 6 (GraphPad Software, La Jolla, CA).

## Supporting Information

S1 Fig Proteasome inhibition sequesters Nipah virus matrix in the nucleus during live virus infection. Extended Focus (maximum intensity projection) view of 3D confocal micrographs of HeLa cells infected with Nipah Malaysia strain at MOI 0.1. DMSO or 1 μM bortezomib was added at 8 h post-infection and cells were fixed at 23 h post-infection. Cells were stained with anti-NiV-M antibodies, green, and counterstained with DAPI to visualize nuclear DNA, blue.
(TIF)

S2 Fig Analysis of the effect of proteasome inhibition on ubiquitination of *Paramyxovirinae* matrix proteins. HEK 293T cells were cotransfected with HA-UbK0 and 3X-Flag tagged **(A)** NiV-M, **(B)** HeV-M, **(C)** SeV-M, **(D)** MuV-M, **(E)** MeV-M or **(F)** NDV-M. At 18 h post-transfection, cells were treated with 10 μM MG132/0.1% DMSO or 0.1% DMSO for 6h. 3X-Flag-tagged-M was immunoprecipitated, and M and ubiquitinated species were detected by immunoblotting against Flag and HA, respectively. The background subtracted integrated fluorescence intensities of the monoubiquitin bands (Ub) normalized to total M (M_0_+M_1_) was determined using LI-COR Odyssey software.
(TIF)

S3 Fig Alanine substitution within the second part of the putative NLS_bp_ of *Paramyxovirinae* matrix proteins. Top, alignments of the NLS_bp_ in WT and bp2-mutants of GFP-tagged NiV-M, HeV-M, SeV-M, MuV-M, NDV-M and MeV-M. Residues mutated to alanines are underlined in blue. Bottom, Extended Focus (maximum intensity projection) views of 3D confocal micrographs of HeLa cells transfected with the WT or bp2-mutant GFP-tagged NiV-M, HeV-M, SeV-M, MuV-M, NDV-M and MeV-M. Cells were counterstained with DAPI to visualize nuclear DNA, blue, and fluorescent phalloidin to visualize the F-actin cytoskeleton, red. Scale bar 10 μm.
(TIF)

S4 Fig Experimental schema and additional controls for ubiquitin-matrix bimolecular fluorescence complementation (BiFC). **(A)** The N and C-terminal fragments of Venus (VN173 and VC155, respectively) are fused to the N-terminus of Ubiquitin (Ub) and viral Matrix (M) proteins as described in Materials and Methods. Covalent conjugation of VN173-Ub to VC155-M by a ubiquitin ligase (E3) brings the spilt Venus fragments (VN173 and VC155) into close proximity to reconstitute a functional Venus fluorophore. The reconstituted fluorescent Venus moiety is stable and essentially irreversible. Thus, the fluorescent Venus tag remains associated with M even if M is subsequently deubiquitinated by a deubiquitinating enzyme (DUB). **(B)** Background controls for ubiquitin-matrix BiFC include mutations in M or ubiquitin that prevent conjugation. **(C)** Extended Focus (maximum intensity projection) view of 3D confocal micrographs of HeLa cells cotransfected with VC155-NiV-M and HA-VN173-Ub or a nonconjugable control, HA-VN173-Ub_K0.G76V_. At 24h post-transfection, cells were counterstained with DAPI to visualize nuclear DNA, blue, anti-HA antibodies to visualize the Ub containing Venus fragment, red, and anti-NiV-M antibodies to visualize the NiV-M containing Venus fragment, grayscale. BiFC fluorescence is pseudocolored green. The matrix mutations that result in decreased ubiquitin-matrix BiFC are the data shown in Fig. 4A-D. **(D)** BiFC assay performed with VC155-fused WT and K258R NiV-M as described above. The BiFC fluorescence (pseudocolored green) per cell was normalized to the matrix fluorescence (red, anti-NiV-M antibodies) in that cell. This normalized Ub BiFC/Matrix was plotted for each cell population expressing WT or K258R NiV-M (n>30 each). p<0.0001 by Student’s t test. **(E)** Immunoblots of transfected HeLa cell lysates performed exactly as for Fig. 4E-H, except that polyclonal anti-NiV-M was used to detect VC-155-fused WT or K258R NiV-M instead of anti-VC155.
(TIF)

S5 Fig Rescue of recombinant GFP-reporter Mumps virus bearing matrix nuclear export mutants. Fluorescence and phase contrast wide-field micrographs of BSRT7 cells at day 4 and 8 post rescue of rMuV-eGFP containing WT, L106A, or K261R mutant MuV-M.
(TIF)

S6 Fig Experimental and computational schemata for evaluating the specificity of protein-protein interactions identified using MudPIT analyses. Left: 3X-Flag-M affinity purification and mass spectrometry (AP-MS) identification of proteins. Nonspecific proteins identified in 3 independent negative-control AP-MS experiments were removed from the list of proteins identified in the 3X-Flag-M AP-MS to generate the putative M interactome presented in Worksheet 1 of each Supplementary Table. Right: Comparison of the putative M interactomes to 21 historic negative-control experiments from the CRAPome mass spectrometry contaminant repository are shown in Worksheet 2 of each Supplementary Table. Those proteins present in the putative M interactomes but seldom found as sources of background contamination in the CRAPome database are considered the most promising for further protein-protein interaction and functional studies.
(TIF)

S7 Fig Interaction of matrix proteins with ubiquitin ligases by coimmunoprecipitation and immunoblot analysis. Anti-flag co-IP from transfected HEK 293T cells as described in Materials and Methods confirming the interaction of **(A)** NiV-M with RAD18 **(B)** NiV-M and NDV-M with UBE2O, **(C)** NiV-M, HeV-M and SeV-M with various Cullin ring ligases, and **(D)** NiV-M and HeV-M, but not SeV-M, with the Cul1 adaptor FBXW11.
(TIF)

S8 Fig Interaction of NiV-M with α-importins and CRM1 by coimmunoprecipitation and immunoblot analysis. Anti-Flag co-IP from transfected HEK 293T cells as described in Materials and Methods confirming the interaction of **(A)** NiV-M with multiple Flag-tagged α-importins and **(B)** NiV-M with 3X-Flag-CRM1.
(TIF)

S9 Fig Overexpressed UBF-1 sequesters NiV-M in the nucleus and inhibits the budding of virus like particles. **(A)** Anti-Flag co-IP from HEK 293T cells transfected with 3X-Flag-NiV-M and GFP-UBF-1. **(B)** XYZ Planes View of 3D confocal micrographs of HeLa cells transfected with GFP-UBF-1 and/or mCherry-NiV-M. Scale bar 10 μm. **(C)** Relative budding of NiV-M with increasing expression of GFP-UBF-1.
(TIF)

S10 Fig Nipah virus matrix localizes to nucleoli during live virus infection. **(A)** Extended Focus (maximum intensity projection) view of 3D confocal micrographs of HeLa cells infected with Nipah Malaysia strain at MOI 10. Cells were fixed with 10% formalin at the indicated time point and stained with anti-NiV-M antibodies, green, and counterstained with DAPI to visualize nuclear DNA, blue. Note prominent nuclear localization at 12 h post-infection. **(B)** XYZ Planes View of a 3D confocal micrograph of HeLa cells infected with Nipah Malaysia strain at MOI 10 for 12 hours. Cells were stained with anti-NiV-M antibodies, green, and counterstained with anti-nucleolin antibodies to visualize nucleoli, red, and with DAPI to visualize nuclear DNA, blue.
(TIF)

S1 Table Putative Nipah virus matrix interacting proteins identified by MudPIT analysis. (XLSX)

S2 Table Putative Hendra virus matrix interacting proteins identified by MudPIT analysis. (XLSX)

S3 Table Putative Sendai virus matrix interacting proteins identified by MudPIT analysis. (XLSX)

S4 Table Putative Newcastle disease virus matrix interacting proteins identified by MudPIT analysis. (XLSX)
