# Supplementary figures and images for: Evidence for Ubiquitin-Regulated Nuclear and Subnuclear Trafficking among Paramyxovirinae Matrix Proteins
Source: PLoS Pathog. 2015 Mar 17;11(3):e1004739. doi: 10.1371/journal.ppat.1004739 (PMC4363627; doi:10.1371/journal.ppat.1004739)

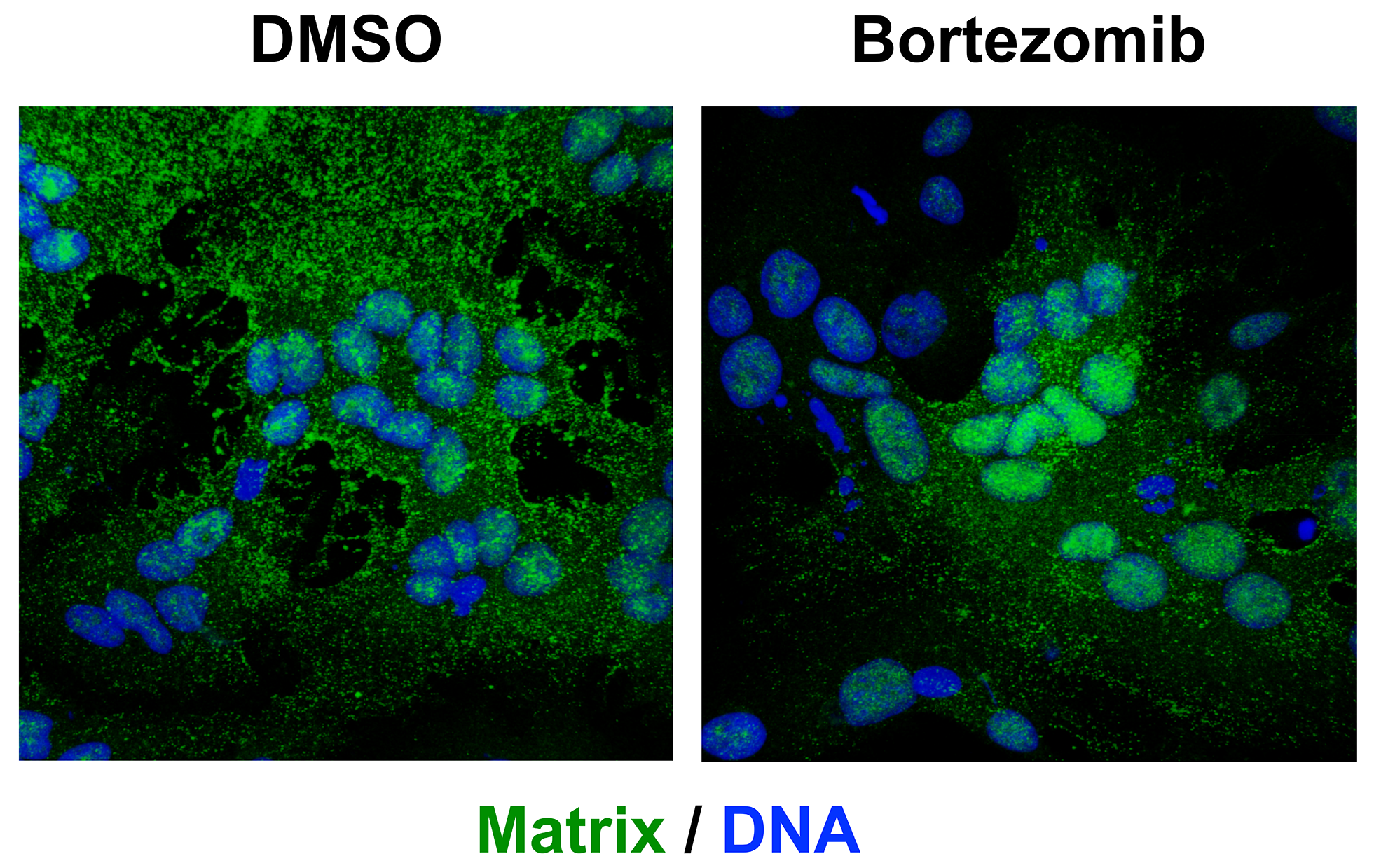

Supplement: S1 Fig — Extended Focus (maximum intensity projection) view of 3D confocal micrographs of HeLa cells infected with Nipah Malaysia strain at MOI 0.1. DMSO or 1 μM bortezomib was added at 8 h post-infection and cells were fixed at 23 h post-infection. Cells were stained with anti-NiV-M antibodies, green, and counterstained with DAPI to visualize nuclear DNA, blue. (TIF) [file ppat.1004739.s001.tif]

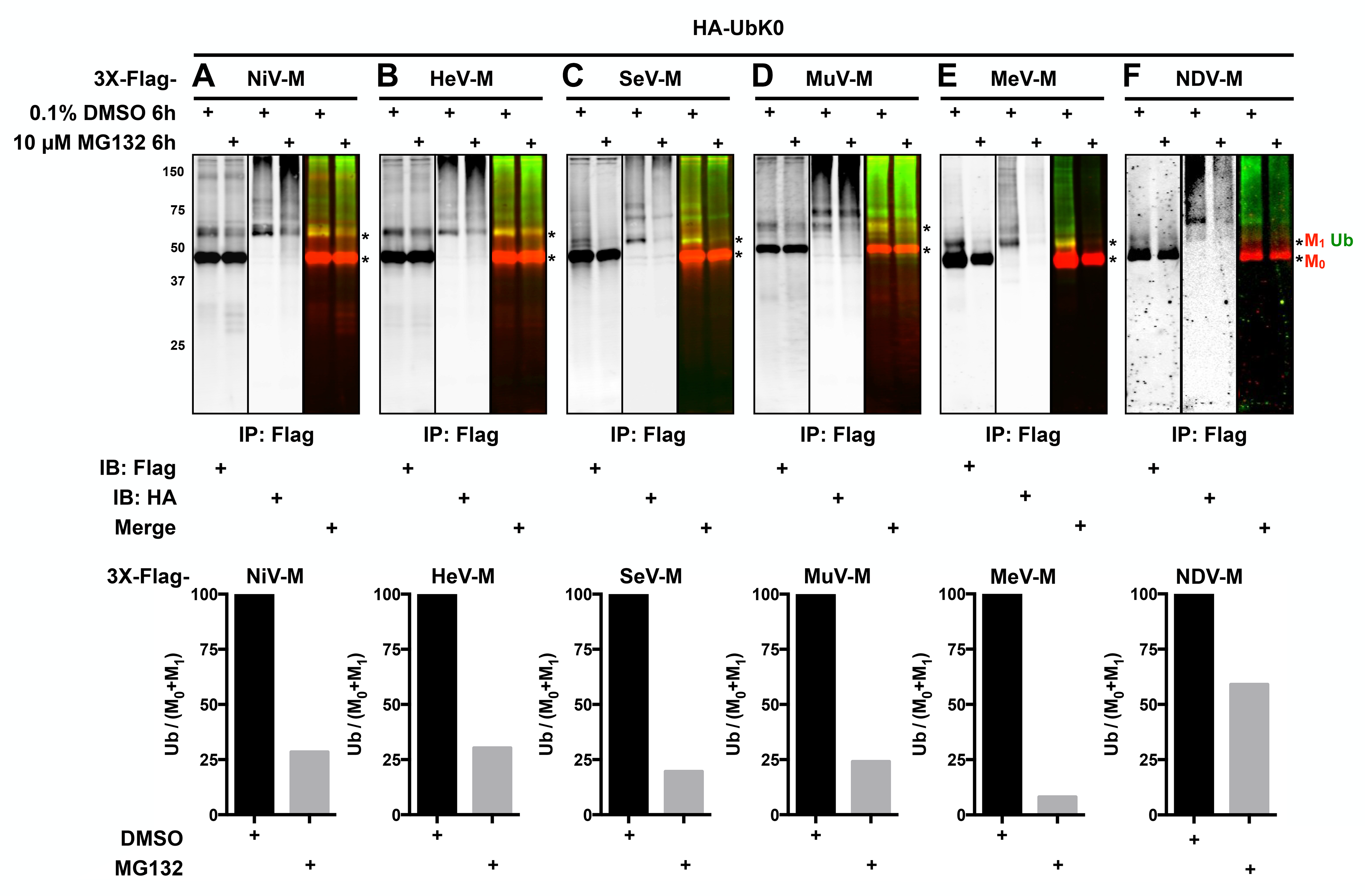

Supplement: S2 Fig — HEK 293T cells were cotransfected with HA-UbK0 and 3X-Flag tagged (A) NiV-M, (B) HeV-M, (C) SeV-M, (D) MuV-M, (E) MeV-M or (F) NDV-M. At 18 h post-transfection, cells were treated with 10 μM MG132/0.1% DMSO or 0.1% DMSO for 6h. 3X-Flag-tagged-M was immunoprecipitated, and M and ubiquitinated species were detected by immunoblotting against Flag and HA, respectively. The background subtracted integrated fluorescence intensities of the monoubiquitin bands (Ub) normalized to total M (M0+M1) was determined using LI-COR Odyssey software. (TIF) [file ppat.1004739.s002.tif]

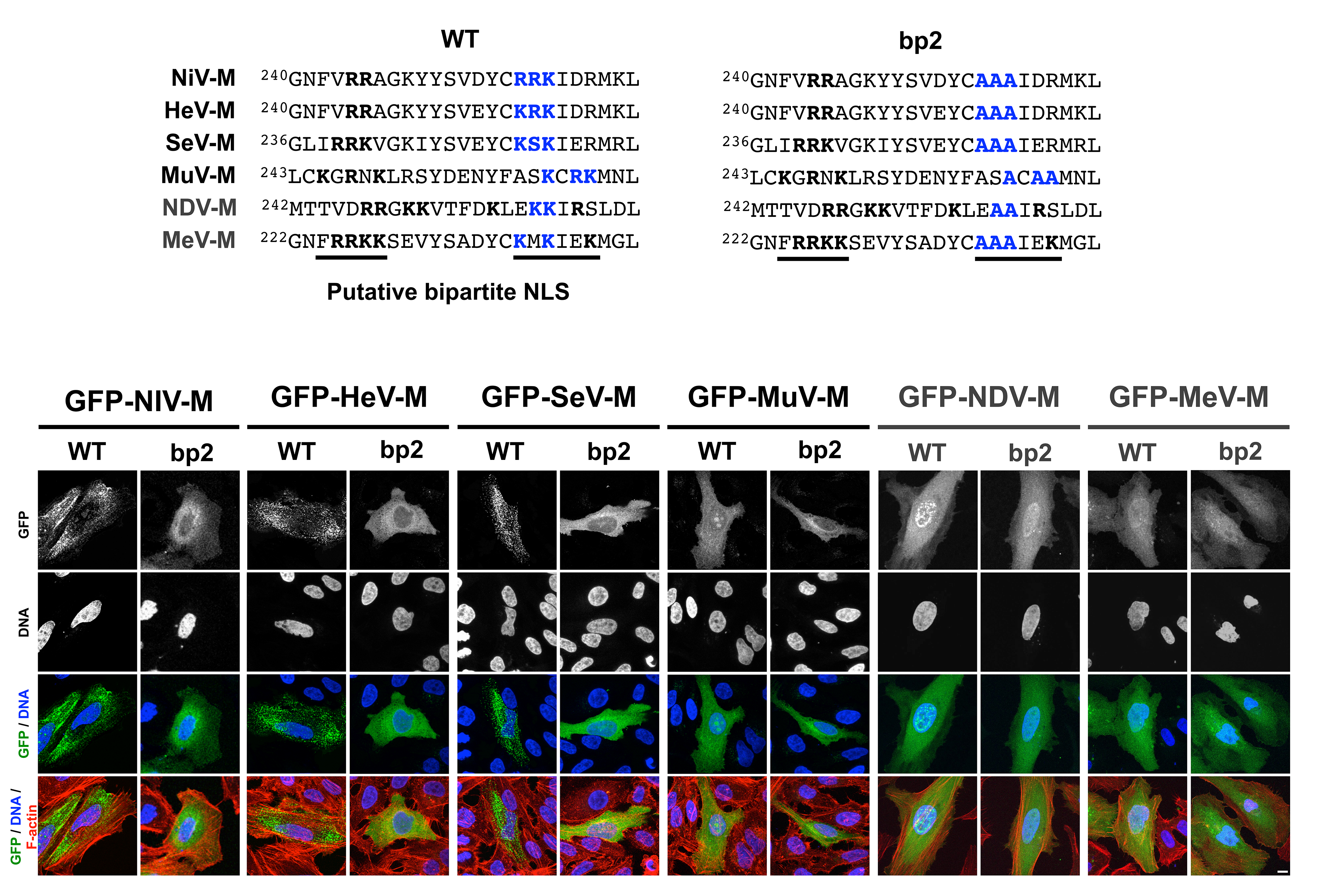

Supplement: S3 Fig — Top, alignments of the NLSbp in WT and bp2-mutants of GFP-tagged NiV-M, HeV-M, SeV-M, MuV-M, NDV-M and MeV-M. Residues mutated to alanines are underlined in blue. Bottom, Extended Focus (maximum intensity projection) views of 3D confocal micrographs of HeLa cells transfected with the WT or bp2-mutant GFP-tagged NiV-M, HeV-M, SeV-M, MuV-M, NDV-M and MeV-M. Cells were counterstained with DAPI to visualize nuclear DNA, blue, and fluorescent phalloidin to visualize the F-actin cytoskeleton, red. Scale bar 10 μm. (TIF) [file ppat.1004739.s003.tif]

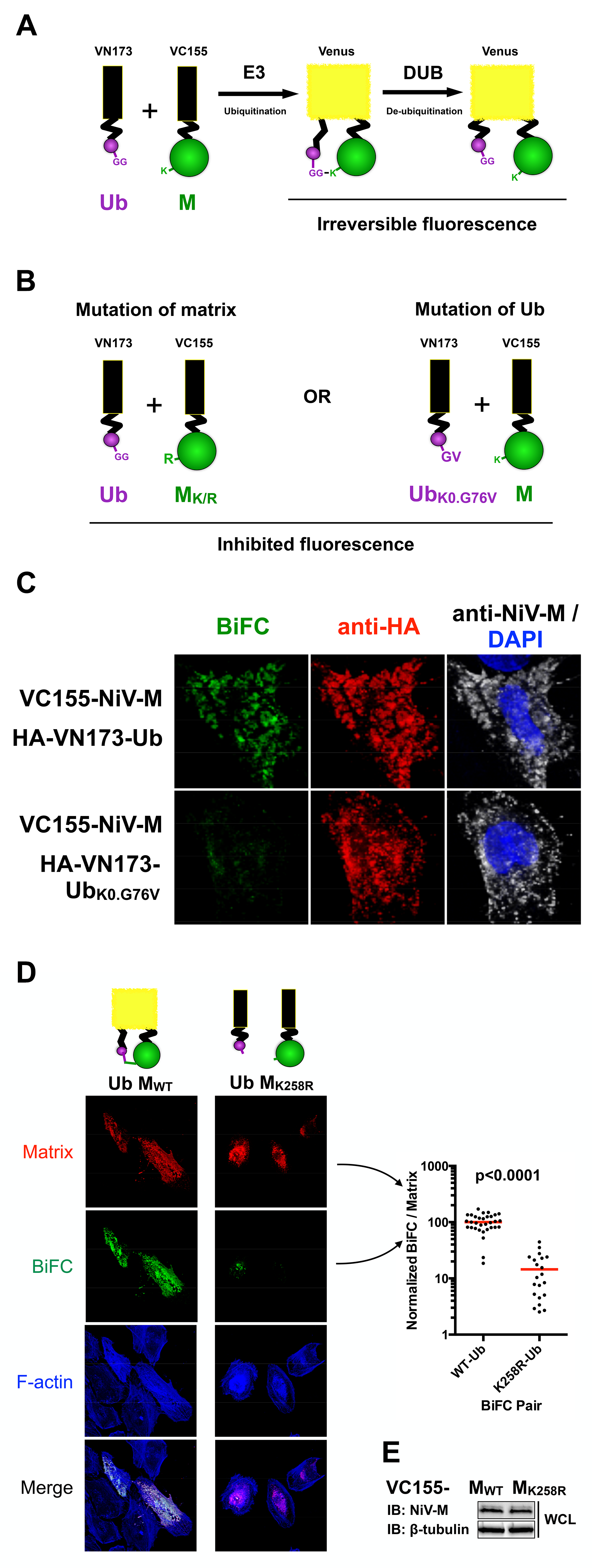

Supplement: S4 Fig — (A) The N and C-terminal fragments of Venus (VN173 and VC155, respectively) are fused to the N-terminus of Ubiquitin (Ub) and viral Matrix (M) proteins as described in Materials and Methods. Covalent conjugation of VN173-Ub to VC155-M by a ubiquitin ligase (E3) brings the spilt Venus fragments (VN173 and VC155) into close proximity to reconstitute a functional Venus fluorophore. The reconstituted fluorescent Venus moiety is stable and essentially irreversible. Thus, the fluorescent Venus tag remains associated with M even if M is subsequently deubiquitinated by a deubiquitinating enzyme (DUB). (B) Background controls for ubiquitin-matrix BiFC include mutations in M or ubiquitin that prevent conjugation. (C) Extended Focus (maximum intensity projection) view of 3D confocal micrographs of HeLa cells cotransfected with VC155-NiV-M and HA-VN173-Ub or a nonconjugable control, HA-VN173-UbK0.G76V. At 24h post-transfection, cells were counterstained with DAPI to visualize nuclear DNA, blue, anti-HA antibodies to visualize the Ub containing Venus fragment, red, and anti-NiV-M antibodies to visualize the NiV-M containing Venus fragment, grayscale. BiFC fluorescence is pseudocolored green. The matrix mutations that result in decreased ubiquitin-matrix BiFC are the data shown in Fig. 4A-D. (D) BiFC assay performed with VC155-fused WT and K258R NiV-M as described above. The BiFC fluorescence (pseudocolored green) per cell was normalized to the matrix fluorescence (red, anti-NiV-M antibodies) in that cell. This normalized Ub BiFC/Matrix was plotted for each cell population expressing WT or K258R NiV-M (n>30 each). p<0.0001 by Student’s t test. (E) Immunoblots of transfected HeLa cell lysates performed exactly as for Fig. 4E-H, except that polyclonal anti-NiV-M was used to detect VC-155-fused WT or K258R NiV-M instead of anti-VC155. (TIF) [file ppat.1004739.s004.tif]

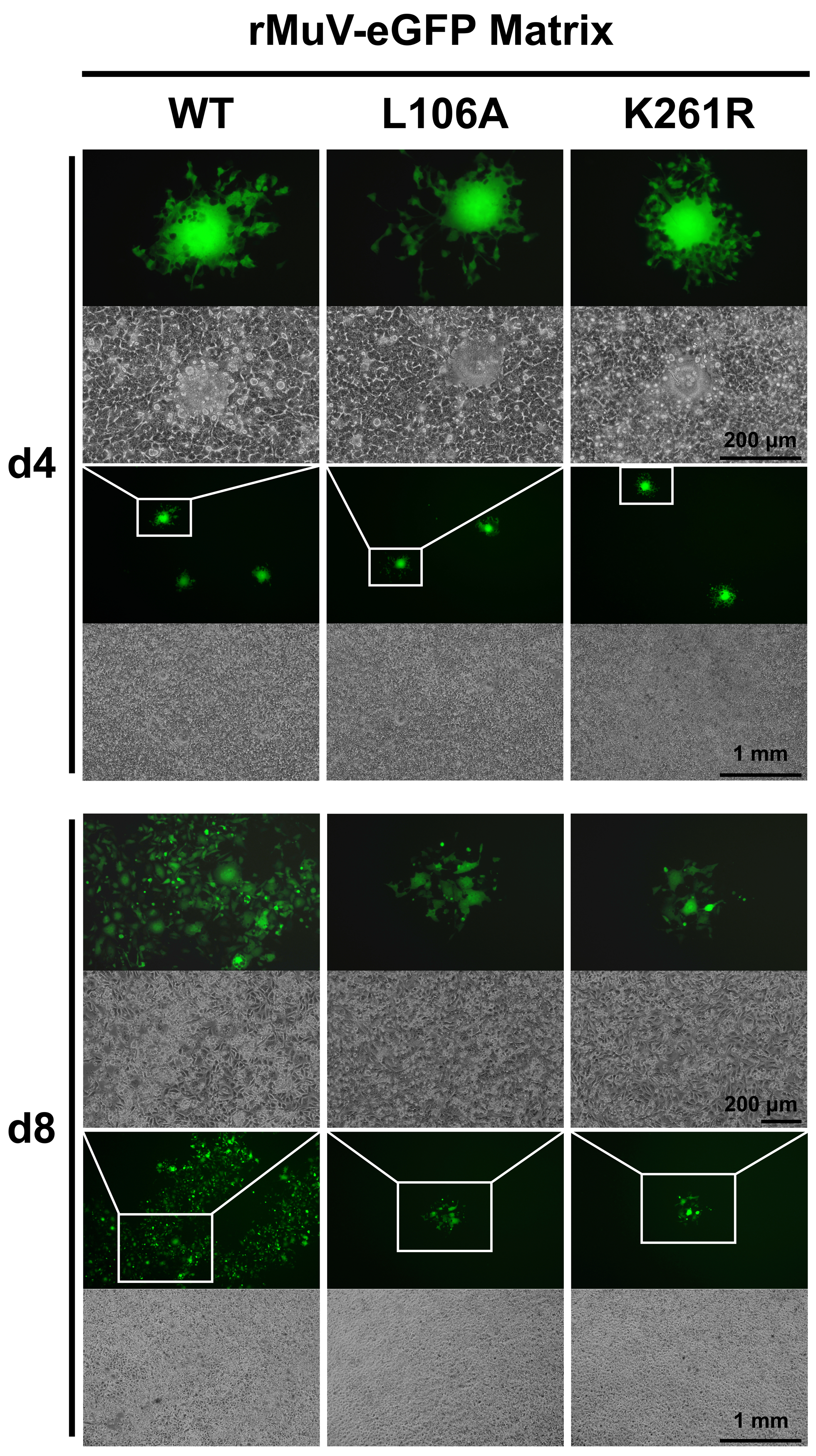

Supplement: S5 Fig — Fluorescence and phase contrast wide-field micrographs of BSRT7 cells at day 4 and 8 post rescue of rMuV-eGFP containing WT, L106A, or K261R mutant MuV-M. (TIF) [file ppat.1004739.s005.tif]

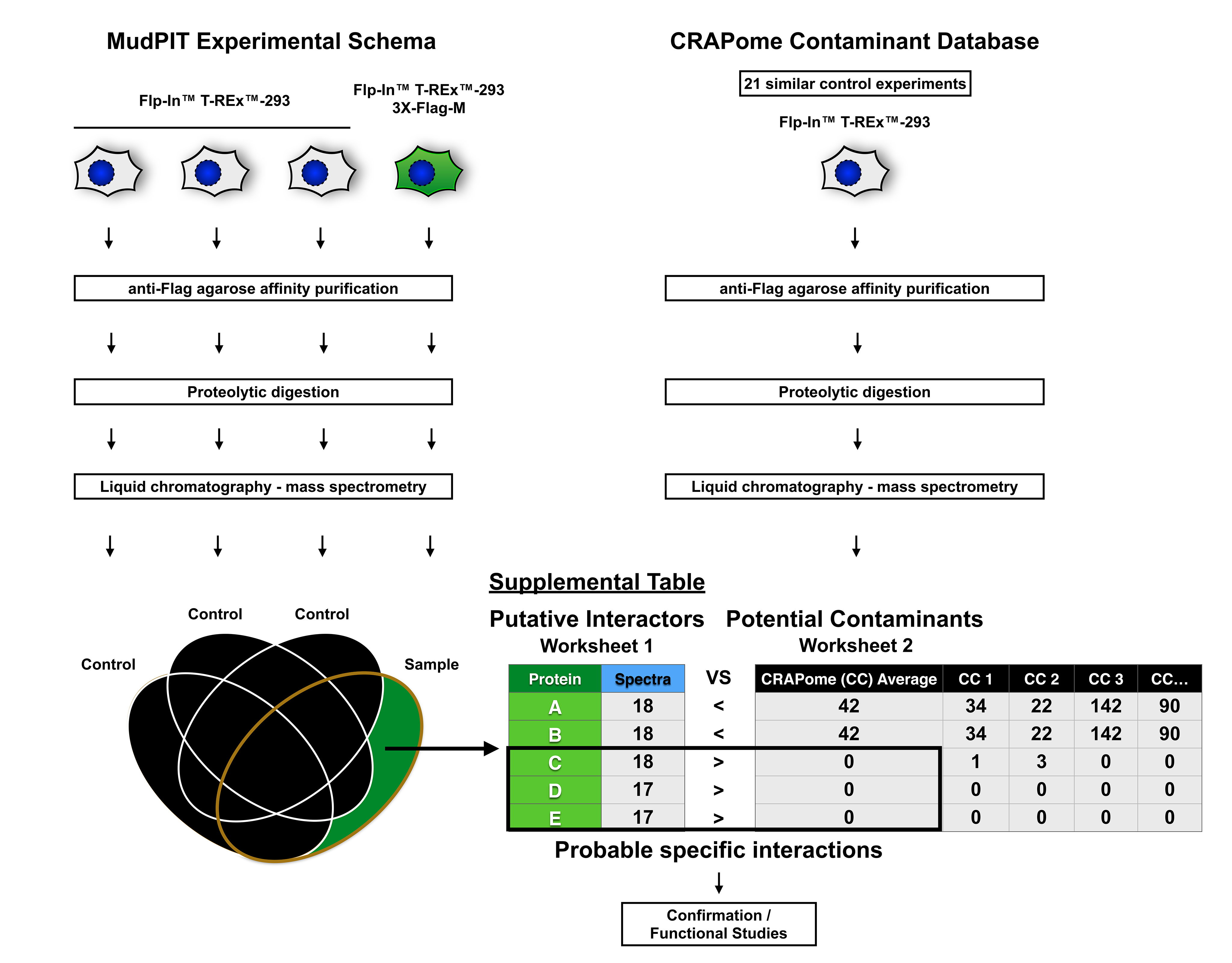

Supplement: S6 Fig — Left: 3X-Flag-M affinity purification and mass spectrometry (AP-MS) identification of proteins. Nonspecific proteins identified in 3 independent negative-control AP-MS experiments were removed from the list of proteins identified in the 3X-Flag-M AP-MS to generate the putative M interactome presented in Worksheet 1 of each Supplementary Table. Right: Comparison of the putative M interactomes to 21 historic negative-control experiments from the CRAPome mass spectrometry contaminant repository are shown in Worksheet 2 of each Supplementary Table. Those proteins present in the putative M interactomes but seldom found as sources of background contamination in the CRAPome database are considered the most promising for further protein-protein interaction and functional studies. (TIF) [file ppat.1004739.s006.tif]

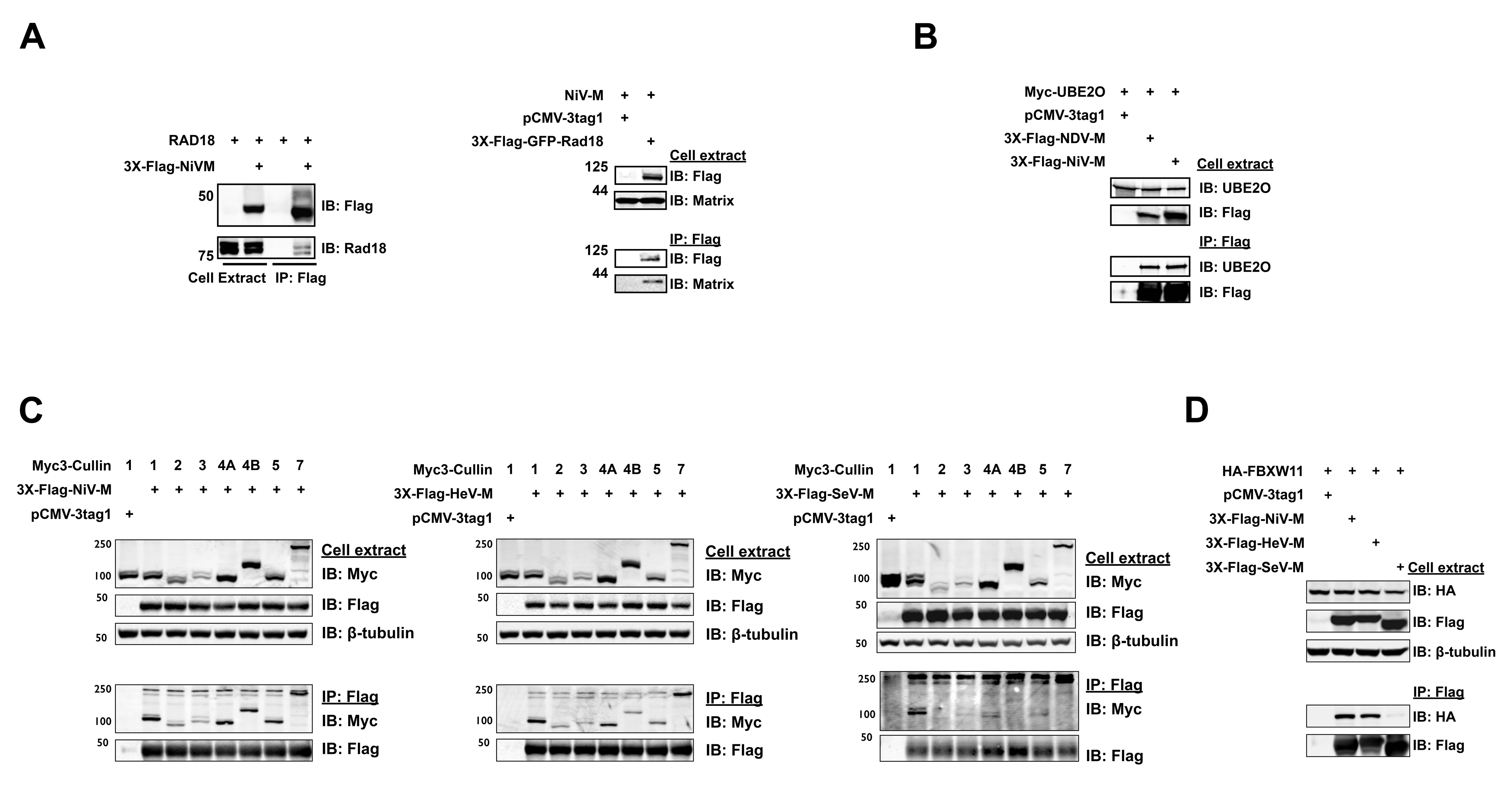

Supplement: S7 Fig — Anti-flag co-IP from transfected HEK 293T cells as described in Materials and Methods confirming the interaction of (A) NiV-M with RAD18 (B) NiV-M and NDV-M with UBE2O, (C) NiV-M, HeV-M and SeV-M with various Cullin ring ligases, and (D) NiV-M and HeV-M, but not SeV-M, with the Cul1 adaptor FBXW11. (TIF) [file ppat.1004739.s007.tif]

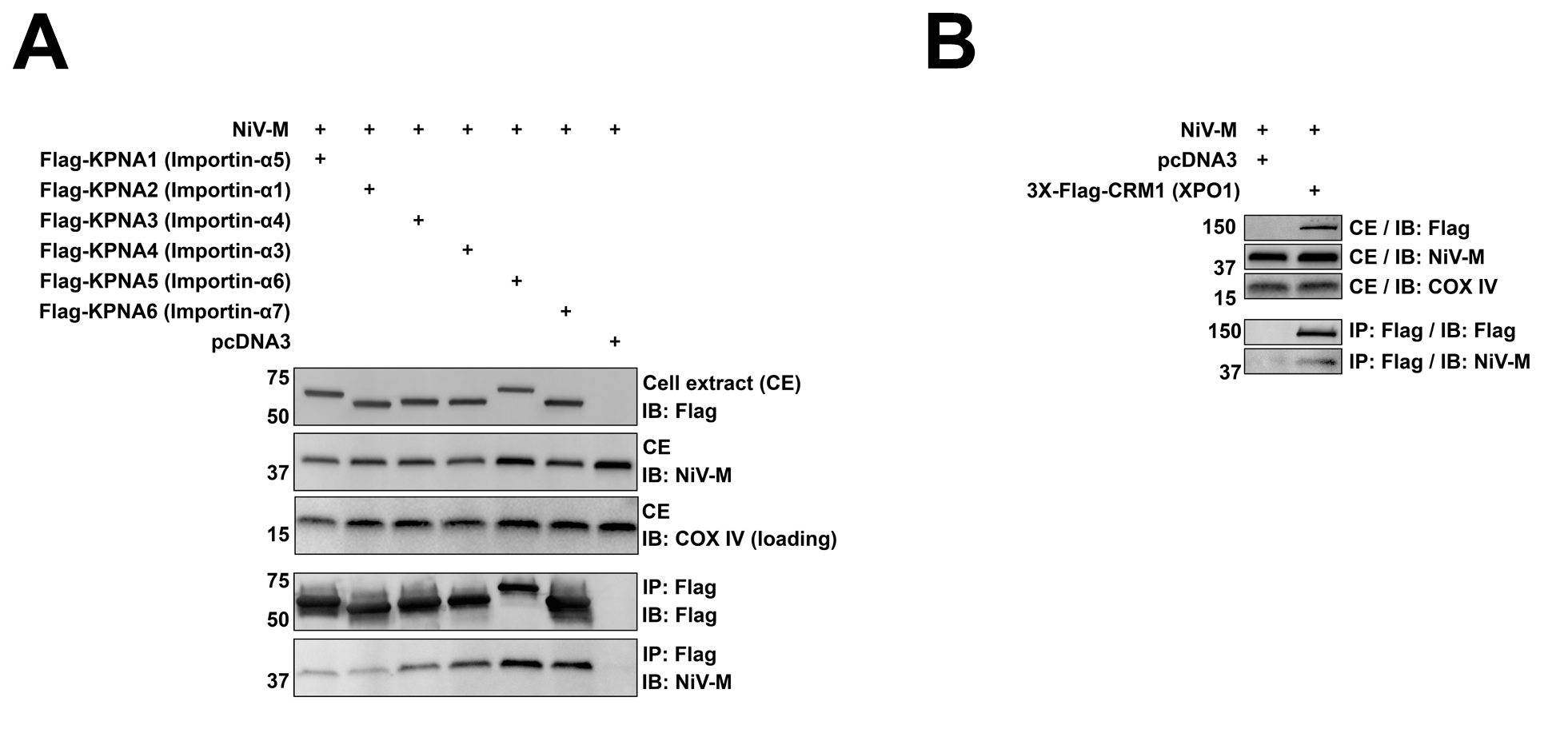

Supplement: S8 Fig — Anti-Flag co-IP from transfected HEK 293T cells as described in Materials and Methods confirming the interaction of (A) NiV-M with multiple Flag-tagged α-importins and (B) NiV-M with 3X-Flag-CRM1. (TIF) [file ppat.1004739.s008.tif]

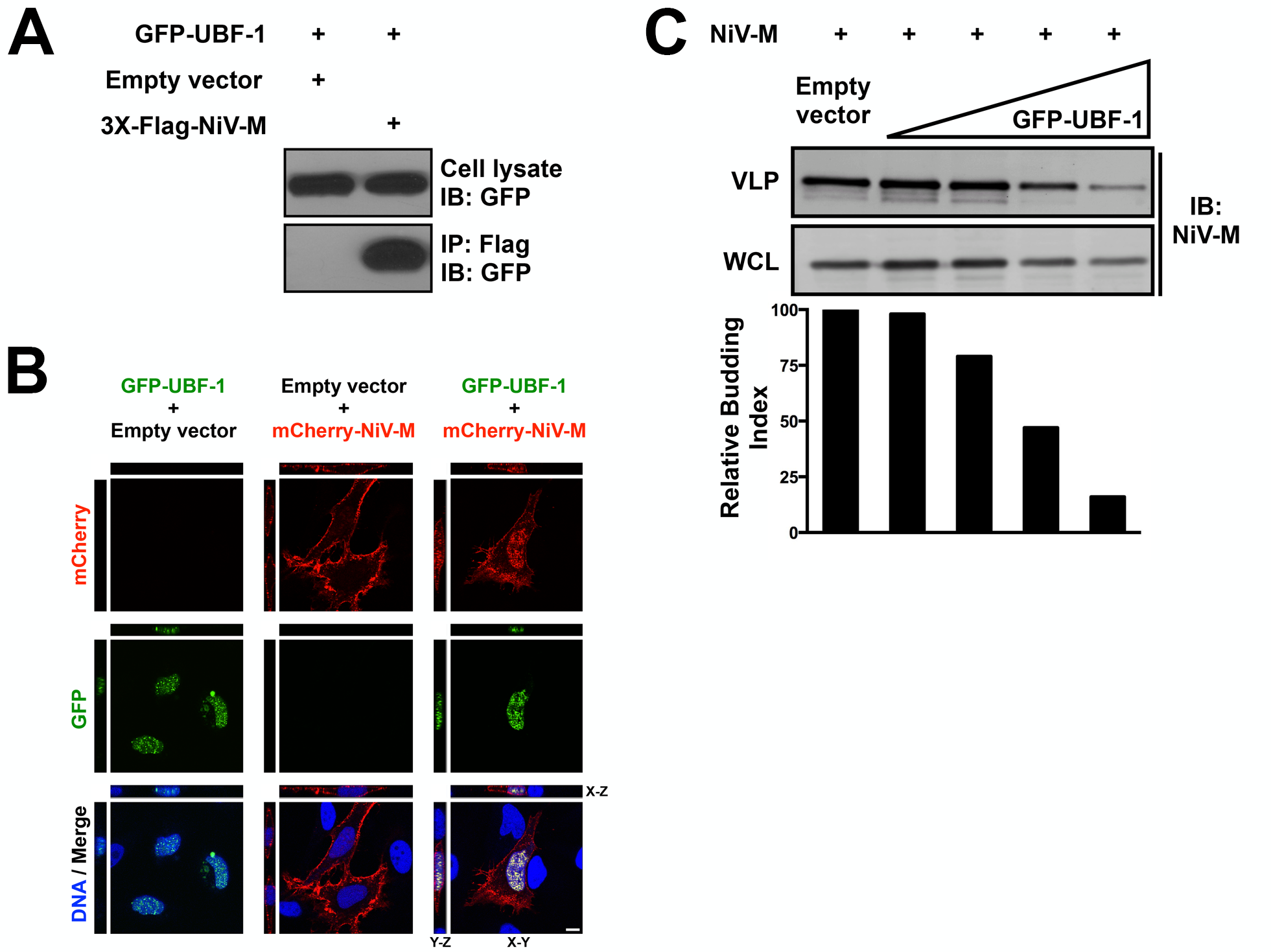

Supplement: S9 Fig — (A) Anti-Flag co-IP from HEK 293T cells transfected with 3X-Flag-NiV-M and GFP-UBF-1. (B) XYZ Planes View of 3D confocal micrographs of HeLa cells transfected with GFP-UBF-1 and/or mCherry-NiV-M. Scale bar 10 μm. (C) Relative budding of NiV-M with increasing expression of GFP-UBF-1. (TIF) [file ppat.1004739.s009.tif]

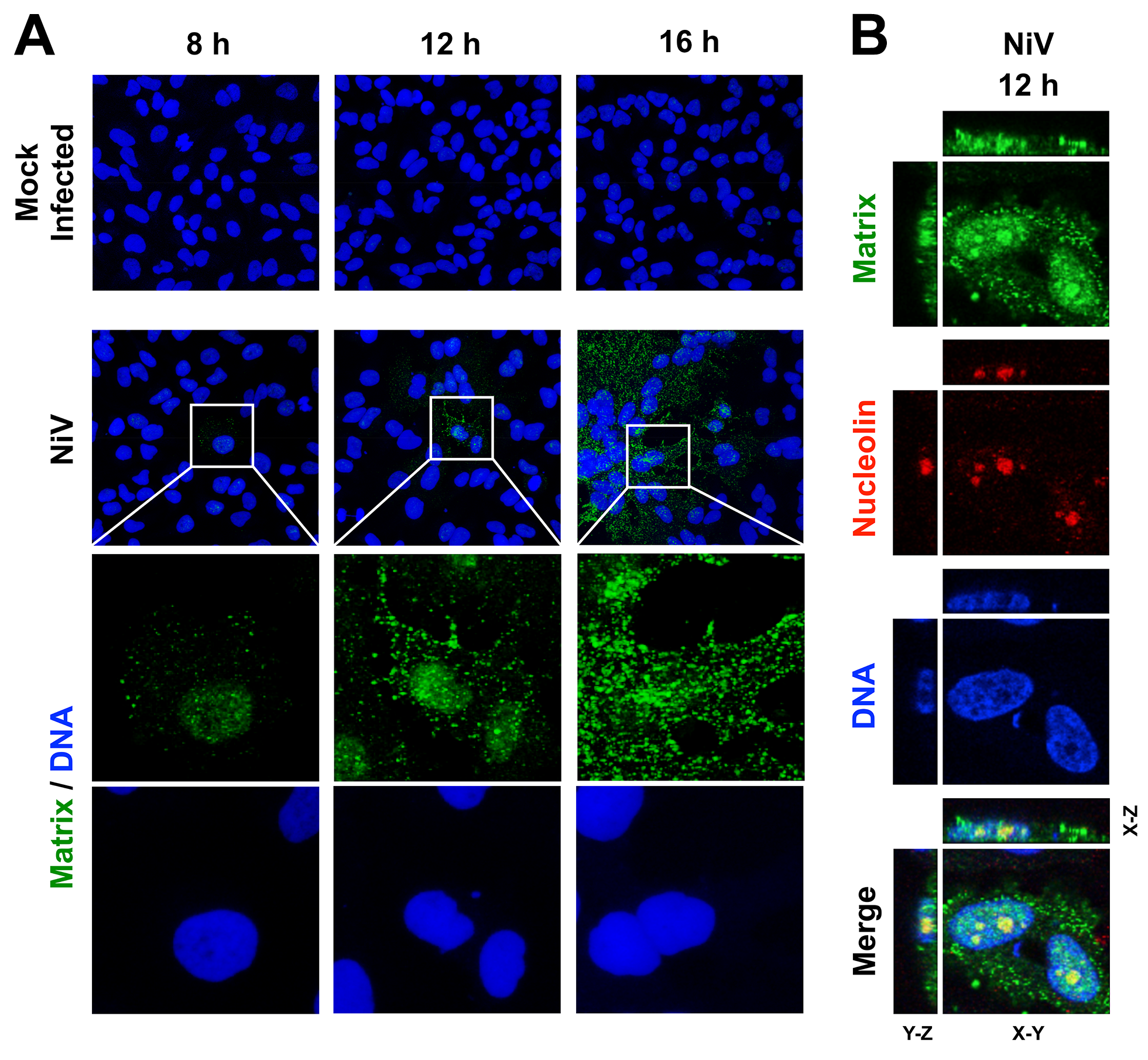

Supplement: S10 Fig — (A) Extended Focus (maximum intensity projection) view of 3D confocal micrographs of HeLa cells infected with Nipah Malaysia strain at MOI 10. Cells were fixed with 10% formalin at the indicated time point and stained with anti-NiV-M antibodies, green, and counterstained with DAPI to visualize nuclear DNA, blue. Note prominent nuclear localization at 12 h post-infection. (B) XYZ Planes View of a 3D confocal micrograph of HeLa cells infected with Nipah Malaysia strain at MOI 10 for 12 hours. Cells were stained with anti-NiV-M antibodies, green, and counterstained with anti-nucleolin antibodies to visualize nucleoli, red, and with DAPI to visualize nuclear DNA, blue. (TIF) [file ppat.1004739.s010.tif]
